# Spatially Confined Co-N_4_ Sites on N-Doped Carbon Nanotube for Efficient Salt-Free Neutral H_2_O_2_ Electrosynthesis

**DOI:** 10.3390/nano16130813

**Published:** 2026-07-01

**Authors:** Manman Zou, Xiaoling Zhuang, Qin Tian, Jili Yuan

**Affiliations:** College of Materials & Metallurgy, Guizhou University, Huaxi District, Guiyang 550025, China13067288995@163.com (X.Z.);

**Keywords:** hydrogen peroxide electrosynthesis, two-electron oxygen reduction reaction, Co-single-atom catalyst, neutral electrolyte

## Abstract

Two-electron oxygen reduction reaction (2e^−^-ORR) represents a sustainable and energy-efficient approach for decentralized hydrogen peroxide (H_2_O_2_) production compared with the conventional anthraquinone process. Among various electrocatalysts, metal–nitrogen–carbon (M–N–C) materials have attracted extensive attention owing to their tunable electronic structures and favorable *OOH adsorption behavior. However, the uncontrolled pyrolysis process generally leads to structurally heterogeneous and ill-defined coordination environments, making it difficult to precisely regulate active sites and understand catalytic mechanisms. Herein, we report a single-atom catalyst (CoN@OCNT) featuring spatially confined pyridinic-N-coordinated Co single sites, synthesized by anchoring a well-defined hexapod terpyridine Co-precursor onto oxidized carbon nanotubes (OCNTs) to suppress metal aggregation during pyrolysis. Benefiting from the optimized coordination environment and enhanced mass/electron transfer, the CoN@OCNT catalyst exhibits nearly 100% H_2_O_2_ selectivity over a wide potential window from −1.0 to 0.66 V versus RHE in neutral electrolyte. In situ FT-IR and Raman spectroscopy reveal a rapid *OOH-mediated reaction pathway during the 2e^−^-ORR process. Furthermore, membrane electrode assembly (MEA) testing demonstrates an H_2_O_2_ production rate of 21.8 mol h^−1^ g_cat_^−1^ with stable operation over 80 h at 60 mA cm^−2^. Remarkably, at an industrially relevant current density of 300 mA cm^−2^, the catalyst achieves a record H_2_O_2_ production rate of 70.3 mol h^−1^ g_cat_^−1^ and a salt-free H_2_O_2_ concentration of 9.4 mM, highlighting its great potential for practical large-scale H_2_O_2_ electrosynthesis in neutral media.

## 1. Introduction

Hydrogen peroxide (H_2_O_2_) is a green and versatile oxidant widely utilized in papermaking, wastewater treatment, sterilization, organic synthesis, and the electronics industry [1,2,3,4]. Currently, over 90% of industrial H_2_O_2_ is produced via the anthraquinone process; however, this mature technology suffers from complicated multistep procedures, high energy consumption, and considerable environmental burden [5,6]. Moreover, the distillation and concentration processes required to obtain highly concentrated H_2_O_2_ introduce substantial safety risks during storage and transportation [7,8,9]. Direct synthesis of H_2_O_2_ from H_2_ and O_2_ has also attracted attention as a potential route for decentralized production, yet its practical implementation remains hindered by costly purification processes and explosion hazards associated with H_2_/O_2_ mixtures [10,11]. For the future of sustainable development, it is extremely important to develop an energy-saving and environmentally friendly on-site H_2_O_2_ synthesis strategy. Therefore, the development of sustainable, energy-efficient, and safe strategies for on-site H_2_O_2_ production is highly desirable. In this regard, the electrochemical two-electron oxygen reduction reaction (2e^−^-ORR) has emerged as one of the most promising alternatives because it enables the green and distributed synthesis of H_2_O_2_ from abundant O_2_ and water under ambient conditions [12]. The realization of efficient 2e^−^-ORR electrosynthesis critically depends on the development of highly active, selective, and low-cost electrocatalysts. To date, substantial advances have been achieved for 2e^−^-ORR in alkaline electrolytes [13,14,15,16]. Nevertheless, H_2_O_2_ readily undergoes disproportionation and decomposition under alkaline conditions, whereas neutral electrolytes provide enhanced H_2_O_2_ stability and offer a direct pathway toward the production of salt-free or low-salt H_2_O_2_ solutions using solid-electrolyte devices [17,18]. Despite these advantages, the sluggish proton-transfer kinetics arising from the low concentrations of H^+^ and OH^−^ in neutral media severely limit catalytic performance, and the development of state-of-the-art neutral 2e^−^-ORR electrocatalysts remains a significant challenge [3].

Metal–nitrogen–carbon (M–N–C) catalysts have emerged as promising platinum-group-metal-free electrocatalysts for 2e^−^-ORR owing to their tunable electronic structures and adjustable active sites through regulation of the metal center and coordination environment, which enables optimized *OOH intermediate binding [8,19,20]. Nevertheless, conventionally synthesized M–N–C catalysts often suffer from structurally heterogeneous coordination environments arising from uncontrollable pyrolysis processes, leading to randomly distributed and poorly defined metal active sites that complicate mechanistic understanding. In addition, maintaining atomically dispersed metal centers generally requires lowering the density of active sites [21,22], which inevitably increases mass-transfer resistance and compromises 2e^−^-ORR performance, thereby limiting practical applications [8]. More importantly, practical electrosynthesis of H_2_O_2_ still faces substantial challenges in simultaneously achieving high current density, high Faradaic efficiency, and the high H_2_O_2_ production concentration required for industrial implementation.

Here, we report the construction of a CoN@OCNT single-atom catalyst with a spatial configuration Co-NC coordination structure through pyrolysis of a CoL_6_ of hexapod-type terpyridine with a clear coordination structure as a precursor and anchor it on an OCNT carrier, which solves the problems existing in traditional research. The selectivity of H_2_O_2_ was improved by adjusting the pyrolysis temperature to control the formation of the active center. The CoN@OCNT catalyst has a high onset potential of 0.66 V vs. RHE for the electrosynthesis of H_2_O_2_ in 0.1 M LiClO_4_ electrolyte, and the selectivity of −1.0–0.66 V vs. RHE is nearly 100%. At the same time, in situ FT-IR and in situ Raman demonstrated that it has a fast OOH mediated kinetic pathway in neutral media. Furthermore, the rate of 21.8 mol h^−1^ g_cat_^−1^ and the stability of 80 h were obtained at 60 mA cm^−2^ through the membrane electrode assembly cells (MEA). At 300 mA cm^−2^ industrial current, the instantaneous H_2_O_2_ production rate was as high as 70.3 mol h^−1^ g_cat_^−1^, and its salt-free H_2_O_2_ concentration was up to 9.4 mM, showing an industrial production rate.

## 2. Materials and Methods

### 2.1. Chemicals and Materials

Commercial multi-walled CNT (short, >50 nm) were purchased from XFNANO (Nanjing, China). Co(NO_3_)_2_•6H_2_O (99%), LiClO_4_ (99%), Na_2_SO_4_ (99%), KMnO_4_ (99.5%), and N,N-dimethylformamide (DMF) were bought from Aladdin Co., Ltd. (Shanghai, China). H_2_O_2_ (30 wt%), CH_3_OH (99%), Isopropanol (99%) and CH_3_CH_2_OH (99.5%) were purchased from Sinopharm Group Chemical Reagent Co., Lt, (Shanghai, China). Proton exchange membrane (Nafion 117) and Nafion solution (5 wt%) were purchased from Du Pont Co., Ltd. (Shanghai, China) Teflon-treated carbon fiber paper (GDS 2230) served as the support of GDE was provided by Sci Materials Hub (http://www.scimaterials.cn/). All aqueous solutions were prepared using deionized water (≥18.25 MΩ).

### 2.2. Catalyst Preparation

The OCNT was prepared by oxidation of concentrations nitric acid on the surface of the original multi-walled CNT. In a typical synthesis, 2 g multi-walled CNT was uniform dispersion in 100 mL HNO3 (68 wt%) under continuous magnetic stirring. The reaction mixture was then heated to 110 °C for 24 h, followed by water washing and vacuum drying to give the resultant OCNT. The CoN@OCNT catalysts were prepared using the impregnation method that CoL_6_ adsorption on the OCNT surface at room temperature. First, 100 mg of OCNT or CNT was added to the 30 mL of DMF solution, labeled solution 1. Meanwhile, a calculated amount of CoL_6_ molecules (0, 5, 10, 20 wt%, compared to OCNT or CNT loadings) was dissolved in the 20 mL of DMF, which is referred to as solution 2. Both solutions 1 and 2 were ultrasonically treated for at least 30 min to disperse OCNT and CoL_6_ in the DMF solution. Then, solution 2 was slowly added to solution 1 under stirring conditions, and the obtained mixed solution was violently stirred at room temperature for 24 h. The powder was obtained by vacuum drying after stirring and filtration. The final catalyst CoN@OCNT was obtained by raising the temperature from 10 °C to 700 °C in the Ar atmosphere and the the temperature was maintained for 2 h. The carbon matrix or precursor, comprising CNT, L_6_ and Co(NO)_3_•6H_2_O, was used to obtain Co@OCNT and N@OCNT, respectively. The product for changing the pyrolysis temperature was named CoN@OCNT-XX; at 600 °C, this converts to CoN@OCNT-600. CoL_6_ and OCNT were mixed and pyrolyzed to obtain CoN@OCNT-GX.

### 2.3. Material Characterizations

The morphology of the material was observed by SEM (JEOL JMS-7500F) and TEM (JEOL JEM-F200). The chemical constituents of the catalysts were identified by XPS (Thermo Fisher Scientific K-Alpha) and FT-IR (Thermo Fisher Scientific Nicolet iS50). The phase structure of catalysts was measured by Raman (LabRam Odyssey with laser excitation at 532 nm) and XRD (Bruker D8 Advance). The Co loadings of CoN@OCNT were detected by XPS. XAFS spectroscopy was carried out using the RapidXAFS 2M (Anhui Absorption Spectroscopy Analysis Instrument Co., Ltd.) by transmission or fluorescence mode at 20 kV and 20 mA, and the Si (533) spherically bent crystal analyzer with a radius of curvature of 500 mm was used for Co.

### 2.4. Electrochemical ORR Measurement

The 2e^–^ ORR performance of CoN@OCNT was studied using an RRDE device. First, 5 mg of catalyst powder was dispersed in the solution that consists of 980 μL DI water, 980 μL isopropanol and 40 μL of 5 wt% Nafion via 30 min ultrasonication for the catalyst ink. The three-electrode system was constructed by coupling the Ag/AgCl reference electrode and Pt counter electrode with RRDE. The Pt ring collector current efficiency N is 0.39. Based on disk current and ring current, the H_2_O_2_ selectivity (FE%) and electron transfer number (n) are calculated by Equations (1) and (2):

(1) $$H_{2}O_{2}\%\;=\;200\;\times \;\frac{I_{d}/N}{{I_{d}\;+\;I}_{r}/N}$$

(2) $$n=4\;\times \;\frac{I_{d}}{{I_{d}+I}_{r}/N}$$

The kinetic current density (j_k_) was calculated according to the Koutecky–Levich Equation (3) [23,24]:

(3) $$\frac{1}{j}=\frac{1}{j_{K}}+\frac{1}{j_{L}}=\frac{1}{j_{K}}+\left(\frac{1}{0.620\;n\;F{\;C_{0}\;D}^{2/3}\;V^{-1/6}}\right)\omega^{-\frac{1}{2}}$$

where j is the measured disk current density and j_K_ and j_L_ are the kinetic current density and the diffusion-limited current densities. n is the electron transfer number calculated by RRDE and F is the Faraday constant (96,485 C mol^−1^). C_0_ is the volume concentration of oxygen, D is the diffusion coefficient of oxygen, and V is the kinematic viscosity of the electrolyte. C_0_, D and V are 1.2 × 10^−6^ mol·cm^−3^, 1.9 × 10^−5^ cm^2^·s^−1^ and 0.01 cm^2^·s^−1^, respectively. ω is the angular rotation rate of RRDE (rad/s).

The rate of PRR and 2e^–^ ORR is described by the current density Equation (4):

(4) $$j_{\mathrm{PRR}}=\frac{i_{\mathrm{PPR}}}{A_{\mathrm{disk}}}\;\mathrm{and}\;j_{\mathrm{peroxide}}=\frac{i_{\mathrm{ring}}}{N\times A_{\mathrm{disk}}}$$

The i_PRR_ is the measured H_2_O_2_ reduction current, A_disk_ is the disk area, and Iring is the oxidation current at the Pt ring of H_2_O_2_.

### 2.5. Electrode Preparation and H_2_O_2_ Electrosynthesis

The electrode was prepared by loading CoN@OCNT material on GDE. First, 20 mg of CoN@OCNT was dispersed in a solution composed of 19.8 mL of ethanol and 0.2 mL of 5 wt % Nafion, and the catalyst ink was formed by ultrasonication for 30 min. A uniform catalyst reaction layer was prepared by spraying 200 μL of CoN@OCNT ink on GDE. An electrochemical two-electrode system for the production of H_2_O_2_ was constructed by using 500 μg cm^−2^ Pt/C-loaded GDE as a counter electrode and proton exchange membrane for the separation of the cathode and anode. The H_2_O_2_ synthesis was carried out using MEA at the constant currents. A large reactor with a carbon paper working area of 4 cm^2^ was used, in which the cathode side was mixed with 1–40 mL min^−1^ DI water and 200 mL min^−1^ pure O_2_ to prepare salt-free H_2_O_2_. At the same time, 1 M Na_2_SO_4_ was used as the anode solution flow rate to control the cycle at 20 mL min^−1^. The constant current is provided by the CHI 760e electrochemical workstation. The generated H_2_O_2_ concentration was measured by KMnO_4_ chemical titration based on Equations (5) and (6):

(5) $$2KMnO_{4}\;+\;5H_{2}O_{2}\;+\;3H_{2}SO_{4}\;\to \;5O_{2}↑\;+\;2MnSO_{4}\;+{\;K}_{2}SO_{4}\;+\;8H_{2}O$$

(6) $$\mathrm{FE}=\frac{H_{2}O_{2}\left(\mathrm{mol}\;L^{-1}\right)\;\times \;2\;\times \;96485\;\times \;V\;(\mathrm{mL})}{j_{\mathrm{total}}\;(\mathrm{mA})\;\times \;t\;(s)}\;\times \;100\%$$

In a typical operation, the H_2_O_2_ solution from the reactor was added to 10 μL of KMnO_4_ solution to reduce Mn^7+^ to Mn^2+^. The titration was completed by judging the color of the solution from red to colorless [18,25].

### 2.6. In Situ FT-IR Characterizations

In situ FT-IR measurements were made on a Thermo Fisher Scientific Nicolet iS50 with a mercury cadmium telluride (MCT) detector. The working electrode was prepared by coating CoN@OCNT catalyst on a Si prism with a chemically deposited Au layer. The specific method is to drop 20 μL of RRDE test catalyst ink on a silicon prism and dry it naturally to make a working electrode. As shown in Figure S15a, in situ FT-IR measurements are performed in a customized H-Cell reactor with Pt wire and Ag/AgCl electrode as the counter electrode and reference electrodes. In situ FT-IR of electrocatalytic synthesis of H_2_O_2_ at different potential was recorded by continuously bubbling O_2_ in 0.1 M LiClO_4_.

### 2.7. In Situ Raman Characterizations

In situ Raman measurements in this work were conducted using the three-electrode PEEK Raman cell with a 1.5 cm diameter circular quartz window on the CHI 760e potentiostat test in conjunction with a Raman spectrometer (LabRam Odyssey with laser excitation at 532 nm). During the in situ Raman measurements, the distance between the window and the working electrode surface was less than 0.1 mm, and the weakening effect of the solution layer on the device was weakened. The CoN@OCNT ink was dropped on a 1.5 × 1.5 cm^2^ carbon paper (GDS 2230) to prepare a working electrode, and the catalyst reached a high loading of 2 mg cm^−2^ to increase the Raman signal intensity. Pt wire and Ag/AgCl electrode were used as counter electrode and reference electrode, respectively, and the test was carried out in O_2_-saturated 0.1 M LiClO_4_. All potentials in situ measurements were referred to vs. RHE.

## 3. Results and Discussions

### 3.1. Synthesis and Structural Characterization of Catalysts

**Figure 1:**
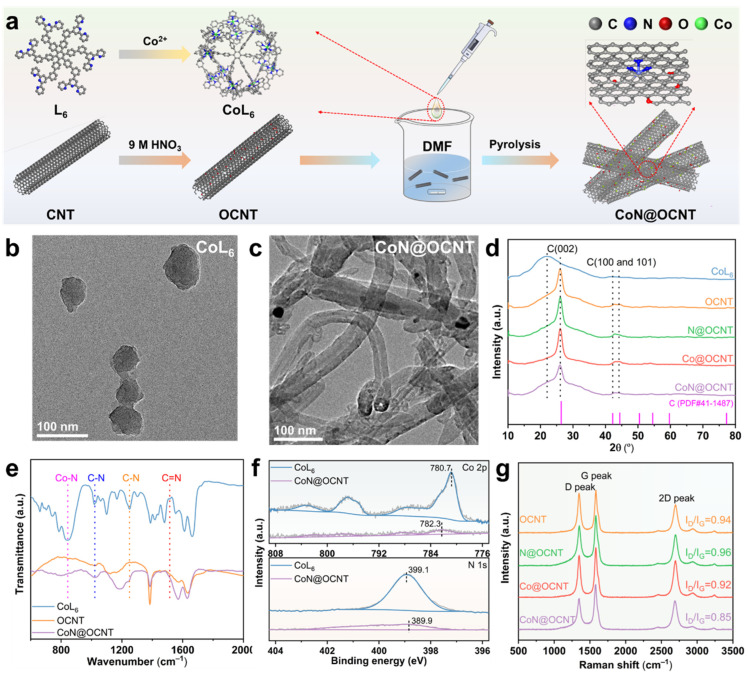
Morphology and structure characterization of the catalysts. (**a**) Illustration of the CoN@OCNT synthesis. (**b**) TEM images of CoL_6_. (**c**) TEM images of CoN@OCNT. (**d**) XRD patterns of CoL_6_, OCNT, N@OCNT, Co@OCNT and CoN@OCNT. (**e**) FT-IR spectra of CoL_6_, OCNT and CoN@OCNT. (**f**) Co 2p high-resolution XPS spectra of CoL_6_, OCNT and CoN@OCNT. (**g**) Raman spectra of OCNT, N@OCNT, Co@OCNT and CoN@OCNT.

As illustrated in Figure 1a, CoN@OCNT was synthesized by combining CoL_6_ with the OCNT support via a sufficient mixing process and anchoring it on OCNT by high-temperature pyrolysis to synthesize a Co-NC catalyst with spatial configuration. The OCNT substrate was prepared by oxidatively treating the original multi-walled carbon nanotubes (CNT) to create a large number of defects. As shown in Figure S1, no obvious change of material morphology was observed before and after the oxidation of CNT. Raman spectra show that OCNT has a significantly enhanced I_D_/I_G_ (0.94) compared to CNT (0.67) due to more carbon defects after activation (Figure S2). In addition, previous studies have indicated that the specific surface area of OCNT will also increase compared to CNT [26], which is beneficial to provide more opportunities to anchor CoL_6_ molecules. The structure and morphology of the catalysts were characterized, as shown in Figure S3; CoL_6_ has a circular nanomaterial structure, while CoN@OCNT mainly has a fibrous structure morphology of OCNT. In the transmission electron microscopy (TEM) images (Figure 1b,c), it is shown that CoL_6_ has a diameter of about 50 nm, and CoN@OCNT only has exposed carbon crystal faces and no obvious metal particle binding HR-TEM is observed (Figure S4). In addition, the element distribution of CoN@OCNT was analyzed by HAADF-STEM and EDS mapping. The results show that C, N, O and Co are evenly distributed around OCNT without obvious Co metal clusters (Figure S5). The N@OCNT, Co@OCNT and Co@CNT prepared by this strategy have similar morphology (Figure S6). These results indicate that OCNT not only improves the dispersion of active sites and inhibits the agglomeration of transition metals, but also, as a carbon substrate, has the advantage of versatility.

X-ray diffraction (XRD) patterns showed that CoN@OCNT-GX catalysts prepared by directly mixed pyrolysis showed two separated carbon crystal plane peaks at 21.0° and 26.3°, suggesting that CoL_6_ and OCNT could not be combined by this method (Figure S7a). The XRD patterns for CoN@OCNT (Figure 1a) show that the characteristic peak at 21.0° was higher than that of OCNT and Co@OCNT, indicating that the post-adsorption pyrolysis strategy successfully anchored CoL_6_ on OCNT and the molecular structure was retained. The FT-IR results of CoN@OCNT show that they are located at 850, 1019, 1252 and 1516 cm^−1^, which belong to the stretching characteristic vibration peaks of Co-N, C-N and C=N (Figure 1e), respectively, being in good agreement with the literature [26,27], and the molecular structure of CoL_6_ anchored on OCNT is further confirmed. Furthermore, X-ray photoelectron spectroscopy (XPS) identifies the chemical elements and composition of CoN@OCNT catalysts. As shown in Figure 1f, the high-resolution Co 2p spectra of CoN@OCNT are mainly associated with Co-N_X_ (782.3 and 797.6 eV) and satellite peaks [28,29]. Table S1 shows that the content of Co is 0.12 wt % with no metal cobalt, which indicates that the Co configuration in CoN@OCNT is more likely to be a single atom characteristic [30,31]. XPS O 1s spectra further suggest that the O element is doped in the carbon nanotube (Figure S7b). In addition, the physical and chemical properties were further studied by Raman spectroscopy. The characteristic D, G and 2D peaks can be observed (Figure 1g) for carbon materials. The I_D_/I_G_ intensity ratios of OCNT, N@OCNT, Co@OCNT and CoN@OCNT were 0.94, 0.96, 0.92 and 0.85, respectively. The results show that the addition of pure L_6_ will cause greater defects. Compared with the low binding of Co ions, on the contrary, CoL_6_ is enriched at the defect position, repairs a large number of defects, and constructs an active center with a single atom. Interestingly, the temperature can adjust this repair process. The I_D_/I_G_ of CoN@OCNT-600 is 0.92 (Figure S8a), which repairs fewer defects and is not enough to construct a large number of coordination structures. As shown in Figure S8b, XRD data can also be observed at 600, and the peak intensity of CoL_6_ at 21.0° is not obvious. In addition, the temperature was further increased to 900 °C, while I_D_/I_G_ increased to 0.99, indicating that a large number of coordination structures were further decomposed. Moreover, the Co 2p XPS peak of CoN@OCNT samples in Figure S8c at 782.2 eV at three temperatures is dominated by Co-N_X_ [28,32]. In addition, the N1s XPS spectra (Figure S8d and Table S3) confirmed that the CoN@OCNT samples contained pyridine-N (398.9 eV), Co-N_X_ (400.0 eV), pyrrole-N (400.8 eV) and graphite-N (401.8 eV) [19,32]. However, the proportion of N was the highest at 700 °C and the pyridine-N content reached 52.9%, indicating that the pyrolysis temperature ensured the stability of the catalyst structure while repairing the defects. Based on these findings, we envision a temperature regulation strategy to control the formation of the CoN@OCNT active center and promote its stable binding to the reaction intermediate to regulate the reactivity of 2e^–^ ORR.

**Figure 2:**
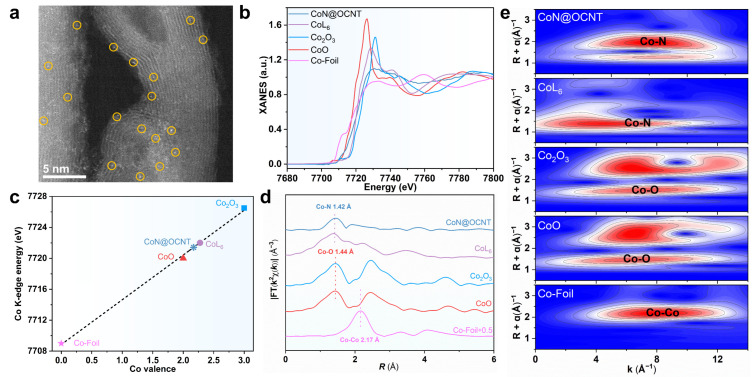
Structure characterization of the CoN@OCNT catalysts. (**a**) HAADF-STEM image of the CoN@OCNT. (**b**) Co K-edge XANES spectra of the CoN@OCNT and reference samples. (**c**) Linear fit of Co valence versus Co K-edge energy position. (**d**) FT k2-weighted and fitting extended XAFS (EXAFS) spectra of the CoN@OCNT and reference samples. (**e**) Wavelet transform (WT) k2-weighted EXAFS contour plots of the CoN@OCNT and reference samples.

The HAADF-STEM measurement is conducted to investigate the Co atom in the CoN@OCNT. As displayed in Figure 2a, the isolated Co bright spots can be observed as uniformly dispersed on the OCNT, which confirms the formation of Co single atom sites. In order to determine the structural configuration of the metal sites in CoN@OCNT, the electronic structures of Co metal centers on CoN@OCNT, CoL_6_, Co_2_O_3_, CoO and Co-Foil were detected by the Co K-edge X-ray absorption near-edge spectroscopy (XANES). As shown in Figure 2b, the near-edge absorption intensity of Co K-edge of CoN@OCNT is between that of Co_2_O_3_ and Co foil, suggesting a positively charged Co atom [26,33]. Further, the linear fitting of the Co valence with the Co K edge energy position demonstrates that the Co valence states of CoL_6_ and CoN@OCNT are +2.26 and +2.18, respectively (Figure 2c). The valence state of Co in CoN@OCNT is slightly lower than that of CoL_6_, showing lower valence binding energy and lower absorption edge energy. We attribute it to the formation of Co-C bond by anchoring CoL_6_ in OCNT. The Fourier transform extended X-ray absorption fine structure (FT-EXAFS) of Co foil indicates that Co exists as an isolated unit point (Figure 2d), and the Co-Co scattering path (~2.17 Å) [23,34] does not exist in CoN@OCNT. The EXAFS fitting of CoN@OCNT shows that there are two main peaks at ~1.29 and ~1.89 Å, which belong to the first scattering path of Co-C and Co-N, respectively [35]. As shown in the WT-EXAFS contour plots (Figure 2e), the samples CoN@OCNT with the Co-C and Co-N coordination structure exhibit an intensity maximum strength at ~6.8 and ~7.3 Å^−1^, which is different than the peak of ~8.4 Å^−1^ of Co-Co bond in Co-Foil. In summary, we have experimentally confirmed that CoN@OCNT has a single-atom catalyst with Co-C and Co-N coordination structures.

### 3.2. ORR Performance and Experimental Investigation

**Figure 3:**
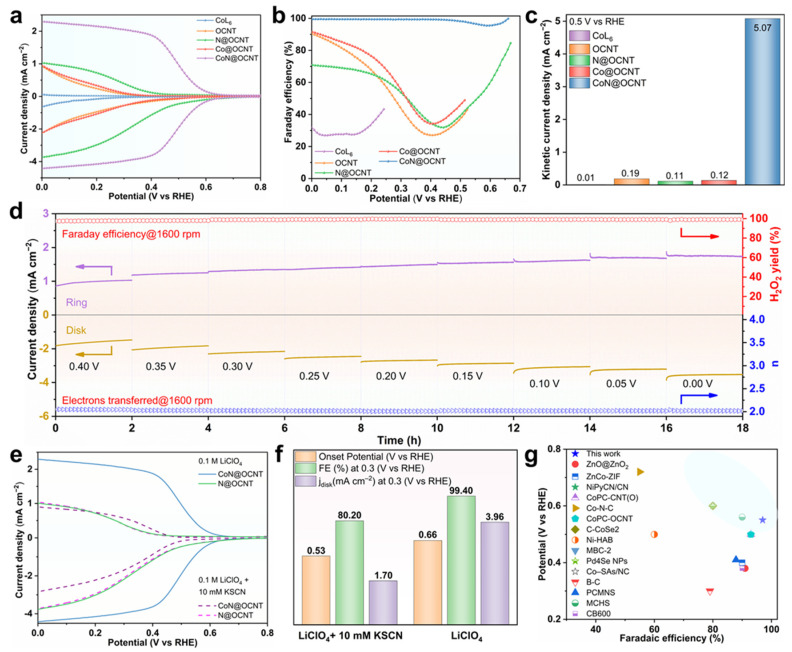
Electrochemical characterization of CoN@OCNT. (**a**) Comparison of LSV curves of different catalysts in O_2_-saturated 0.1 M LiClO_4_ at 1600 rpm. (Upper: ring current densities; bottom: disk current densities). (**b**) H_2_O_2_ FE over the applied potential range 0.0 to 0.7 V. (**c**) Comparison of kinetic current densities measured at 0.5 V. (**d**) The amperometric i-t curve profiles of CoN@OCNT over the potential range from 0.4 to 0.0 V. (**e**) LSV curves in 0.1 M LiClO_4_ before and after the addition of 10 mM SCN^−^. (**f**) The changes of onset potential, FE and n of CoN@OCNT before and after the addition of SCN^−^. (**g**) H_2_O_2_ selectivity of CoN@OCNT and the reported catalysts in neutral media (Table S5) at 1 mA cm^−2^ of j_disk_.

The 2e^–^ ORR activity and selectivity of catalysts were assessed by using a standard three-electrode system rotating ring-disk electrode (RRDE) in 0.1 M LiClO_4_ (pH = 7.0) electrolyte. The catalyst was precisely spin-coated in the disk platinum–carbon region to catalyze ORR to generate H_2_O_2_, and a 1.2 V oxidation potential was applied to the Pt ring electrode to detect the formation of H_2_O_2_. This part of the test does not determine that the potential is relative to the reversible hydrogen electrode. Samples of CoL_6_, OCNT, N@OCNT, Co@OCNT and CoN@OCNT werer compared. The cyclic voltammetry (CV) curve of CoN@OCNT shows that the disk current has an obvious oxygen reduction peak and the ring current has an obvious oxidation peak in O_2_-saturated 0.1 M LiClO_4_ solution, while the redox peaks of CoL_6_ and OCNT are not obvious (Figure S9). The ORR and H_2_O_2_ oxidation current density in Figure 3a exhibit that CoN@OCNT catalysts show better activity with a higher onset potential of 0.66 V (defined at −0.1 mA cm^−2^ of disk current density) [26] compared to CoL_6_ (0.24 V) and OCNT (0.52 V). At the same time, CoN@OCNT has a high ORR current density of 3.61 mA cm^−2^ at 0.4 V. Furthermore, with N@OCNT and Co@OCNT, the excellent oxygen reduction performance of the catalyst combined with CoL_6_ and OCNT was highlighted. Significantly, CoN@OCNT exhibits the highest H_2_O_2_ selectivity of nearly 100% in the potential range of 0–0.50 V (Figure 3b). In contrast, CoL_6_, OCNT, N@OCNT and Co@OCNT showed lower 2e^–^ ORR catalytic selectivity, as indicated by their average selectivity values of 30%, 60%, 63% and 70%, respectively. The number of electrons transferred (n) of CoN@OCNT is calculated to be 2.01–2.00 at 0–0.50 V, which further indicates that it is more inclined to occur 2e^–^ ORR (Figure S10). Combined with the calculated kinetic current, CoN@OCNT has a kinetic current density of 5.07 mA cm^−2^ at 0.5 V, which is significantly higher than other catalysts, further proving that it has high ORR activity (Figure 3c). As shown in Figure S11, CoN@OCNT exhibited a mass current density of 51.4 A g^−1^ at 0.5 V, which is approximately 514 times, 36.7 times, 26.6 times and 44.6 times higher than those of CoL_6_, OCNT, N@OCNT and Co@OCNT, respectively. The Tafel slope of 133 mV dec^−1^ for CoN@OCNT is smaller than other catalysts, indicating faster reaction kinetics of H_2_O_2_ synthesis in the neutral medium for CoN@OCNT [23].

Furthermore, we systematically studied the effect of pyrolysis temperature on the selectivity of H_2_O_2_ electrosynthesis. The catalyst prepared by simply adsorbing CoL_6_ on OCNT has almost no improvement in 2e^−^ ORR activity compared to OCNT in a neutral medium. As the temperature increases to 600 °C, a small amount of Co-C bonds should be formed to repair the defects, which corresponds to Figure S8a, increasing the onset potential by 100 mV. Further enhancing the temperature to 700 °C, it can be proved by Figure 1g Raman data that high temperature promotes the formation of a large number of Co-C bonds at the defect position [30,36,37] and then constructs Co-NC active sites with spatial configuration. The overpotential of CoN@OCNT-800 increased in a neutral medium and the activity of 2e^–^ ORR decreased (Figure S13), indicating that the coordination structure was decomposed and the defects increased (Figure S8a). Interestingly, the activity of CoN@OCNT-900 and OCNT is almost the same, showing that the coordination structure is completely decomposed. As shown in Figure S14, the TOF values of the catalyst at different pyrolysis temperatures were measured at 0.5 V, where Co@OCNT is as high as 78.7 s^−1^. In summary, combined with material characterization and activity test data, it is shown that the pyrolysis temperature can regulate the synthesis of active sites of Co-NC spatial configuration and exhibit excellent 2e^–^ ORR activity. The activity and selectivity of CoN@OCNT-GX were further tested to prove the necessity of the adsorption process. As shown in Figure S15, compared with OCNT, the ORR performance of OCNT did not improve but decreased, indicating that the purpose of preparing Co-NC active center by simple physical mixing cannot be achieved by CoL6 to repair the defects of OCNT. This conclusion can be proved by the increase of Ramam data defects (Figure S7) and the appearance of separated CoL_6_ high-resolution Co 2p XPS (Figure S16).

To systematically study the H_2_O_2_ selectivity for CoN@OCNT, we proceeded with the RRDE testing at the amphoteric i–t curve (i–t) conditions from 0.4 to 0.0 V. As shown in Figure 3d, CoN@OCNT shows that the selectivity of H_2_O_2_ is more than 95% in the potential range of 0.40~0.3 V and nearly to 99% in the range of 0.3~0.0 V. It shows that CoN@OCNT follows a highly selective and stable 2e^–^ ORR pathway in a wide potential range, indicating its potential for scalable electrolysis. Next, we conducted further experimental studies on N@OCNT and CoN@OCNT catalysts to explore the active origin of the 2e^–^ ORR pathway. It was found that the onset potential and selectivity of CoN@OCNT were significantly reduced by 0.13 V and 19.2%, respectively, after the introduction of KSCN poisoning in a neutral medium, and the increase of n to 2.4 improved the selectivity of the 4e^−^ pathway (Figure 3e,f). In addition, the SCN^−^ poisoning experiment showed little change in the activity of the N@OCNT catalyst without Co addition, further confirming the important contribution of the Co center to the 2e^–^ ORR [38,39]. In order to evaluate the 2e^–^ ORR performance of the catalysts more comprehensively and objectively, we compared the catalysts for the preparation of H_2_O_2_ in neutral media in recent years. The CoN@OCNT can have a potential of 0.55 V and a selectivity of 97.0% when the disk current density of 1 mA cm^−2^, which is a previously reported high value for the calibration in a neutral electrolyte (Figure 3g and Table S4). It was further shown that CoN@OCNT has great potential to achieve the required low overpotential neutral H_2_O_2_ electrosynthesis at high current density.

### 3.3. In Situ Characterization of 2e^–^ ORR

**Figure 4:**
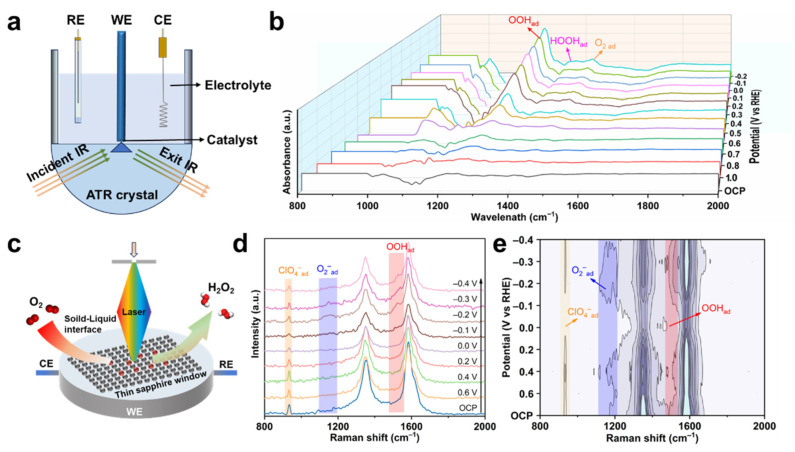
In situ characterization of the ORR catalytic mechanism. (**a**) In situ FTIR device schematic. (**b**) In situ FTIR spectra recorded during electrosynthesis of H_2_O_2_ on CoN@OCNT in O_2_-saturated 0.1 M LiClO_4_ solution (OOH_ad_: adsorbed *OOH, HOOH_ad_: adsorbed *HOOH and O_2 ad_: adsorbed *O_2_). (**c**) In situ Raman device schematic. (**d**) In situ Raman spectra of CoN@OCNT electrocatalysts at selected potentials in O_2_-saturated 0.1 M LiClO_4_ solution. (**e**) Corresponding contour plots of in situ Raman spectra for CoN@OCNT electrocatalyst.

The in situ Fourier transform infrared spectroscopy (in situ FTIR) and in situ Raman spectroscopy (in situ Raman) monitored the interaction between oxygen intermediates and CoN@OCNT during the electrolytic H_2_O_2_ synthesis (Figure 4a,c and Figure S17). For the in situ FTIR spectra, a weak absorption band appeared at 1238 cm^−1^ in addition to 0.7 V potential, and the absorption band gradually increased rapidly as the potential decreased, which corresponds to the ORR activity test data (Figure 3a and Figure 4b). At the same time, two lower absorbance peaks appear at 1396 and 1437 cm^−1^. According to previous reports, these three peaks can be attributed to the adsorption of *OOH (OOH_ad_), *HOOH (HOOH_ad_) and *O_2_ (O_2 ad_) [19,26,40,41]. Compared with previous research values, the wave number may be slightly shifted due to different adsorption positions [26]. In addition, in the in situ Raman spectrum, two bands corresponding to the D peak and G peak can be seen at different applied potentials (Figure 4d). In Figure 4d,c, there are three peaks at 936, 1151 and 1526 cm^−1^, which are attributed to the vibration of the active sites of ClO_4_^−^_ad_, O_2_^−^_ad_ and OOH_ad_ adsorption catalyst, respectively, and the intensity increases with the increase of voltage. Overall, in situ FTIR and in situ Raman detected CoN@OCNT of the potential-dependent adsorbed OOH band in a neutral solution, supporting its rapid *OOH-mediated 2e^–^ ORR pathway.

### 3.4. Practical-Scale Neutral Production of H_2_O_2_ Electrosynthesis

**Figure 5:**
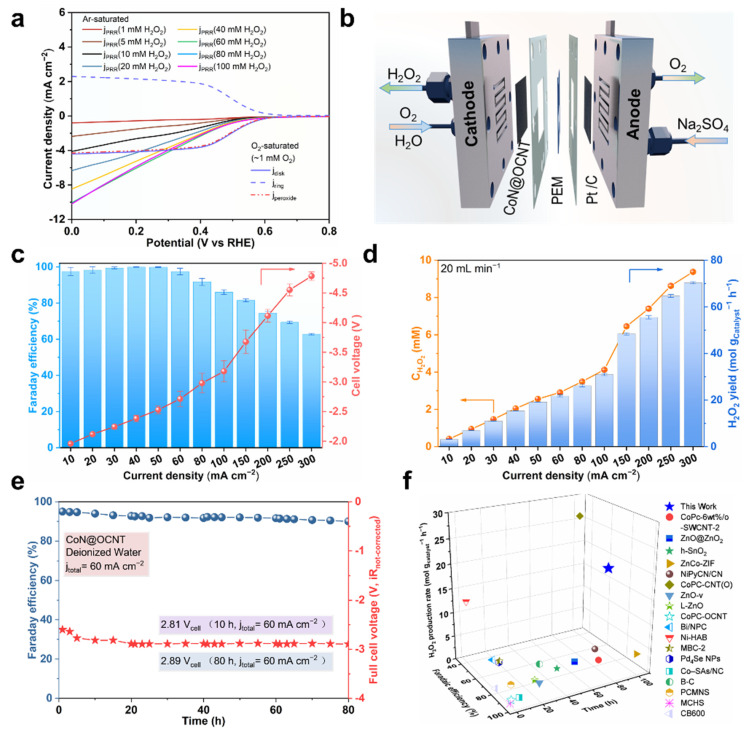
H_2_O_2_ electroproduction in the flow cell. (**a**) In situ ATR-FTIR device schematic. (**a**) RRDE studies of oxygen reduction reaction on CoN@OCNT at 1600 rpm in O_2_-saturated 0.1 M LiClO_4_, in comparison with peroxide reduction reaction (PRR) current densities (j_Peroxide_) at 1600 rpm in Ar-saturated 0.1 M LiClO_4_ containing 1, 5, 10, 20, 40, 60, 80 and 100 mM H_2_O_2_. (**b**) Schematic diagram of the membrane electrode assembly (MEA) systems for H_2_O_2_ production. (**c**) The I–V curve and corresponding FEs for producing H_2_O_2_ using the MEA through flowing DI water and 200 mL min^−1^ O_2_ in the cathode chamber. (**d**) Concentration and yield of H_2_O_2_ in one-pass flowed out of the cathode solution. (**e**) Long-term H_2_O_2_ electrosynthesis at 60 mA cm^−2^ in a two-compartment MEA device. (**f**) The H_2_O_2_ selectivity, H_2_O_2_ yield and stability of CoN@OCNT were compared with those of the current neutral medium catalyst (Table S5) in the scalable electrolytic cell.

The RRDE test only detects instantaneously generated H_2_O_2_ and ignores the H_2_O_2_ concentration in the solution. The thermodynamically generated H_2_O_2_ can be further electroreduced to H_2_O (H_2_O_2_ + 2H^+^ + 2e^–^ → 2H_2_O E^o^ = 1.76 V) [31,42]. As the prepared H_2_O_2_ can accumulate to a practically useful concentration, it is important to evaluate the H_2_O_2_ reaction (PRR) [43]. In this paper, we studied the PRR by connecting the same catalyst-supported CoN@OCNT-coated RRDE to the three-electrode battery in 0.1 M LiClO_4_ solution containing Ar saturated 1–100 mM H_2_O_2_. Figure 5a shows that the PRR and 2e^–^ ORR of CoN@OCNT exhibit similar onset potential. The PRR rate is enhanced with the increase of H_2_O_2_ concentration and overpotential and reaches the maximum at 60 mM H_2_O_2_. The comparison of j_ORR_ and j_PRR_ at different concentrations of H_2_O_2_ provides a theoretical basis for the accumulation of H_2_O_2_, in which the net growth rate of H_2_O_2_ remains positive in the range of 0.33–0.60 V (Figure S18). Furthermore, CoN@OCNT shows an upward trend in current density over a wide potential range from −1.0 V to 0.66, and the selectivity of H_2_O_2_ is 95–100% and the value of n is in the range of 2.0–2.2 (Figure S19), which confirms that CoN@OCNT has great application potential in the industrial-related yield of neutral H_2_O_2_ production under the condition of O_2_ supply and high current density.

Inspired by the excellent 2e^–^ ORR performance of CoN@OCNT, to evaluate the production capacity of H_2_O_2_, the catalyst was sprayed on the gas diffusion electrode to increase the oxygen supply. We assembled custom flow membrane electrode assembly cells (MEAs) using a two-electrode system, as illustrated in Figure 5b. The MEAs cathode can effectively synthesize H_2_O_2_ molecules from O_2_ and DI water, and the H^+^ generated by the oxygen evolution reaction of the anode Pt/C penetrates the Nafion member into the cathode, ensuring that H^+^ compensates for the charge and also reduces the flow of Na+ [18]. As shown in Figure S20, the cell pressure and H_2_O_2_ selectivity increase with increasing flow rate at a current density of 50 mA cm^−2^, and reach nearly 100% at 20 mL min^−1^, indicating that increasing the flow rate of DI water is helpful to take out the electrosynthesized H_2_O_2_. Further studies under the same conditions of 100 μg cm^−2^ showed that the selectivity and activity of H_2_O_2_ increased with the increase in flow rate, but the activity was lower than that of 50 μg cm^−2^ (Figure S21). Figure S22 combines RRDE to study the effect of different CoN@OCNT loadings. It can be proved that although the increase of loading will increase the activity of H_2_O_2_, it will lead to the decrease of H_2_O_2_ selectivity and mass activity, and the calculated n is more deviated from 2e^–^ ORR. The ORR of CoN@OCNT started at a cell voltage of 1.5 V in MEA and reached an industrial current density of 300 mA cm^−2^ at 4.0 V (Figure S23). At the same time, the effect of current density on selectivity and cell voltage was studied (Figure 5c). The selectivity was close to 100% at 10–50 mA cm^−2^ and 95% selectivity and 21.8 mol h^−1^ g_cat_^−1^ activity were obtained at 60 mA cm^−2^. Moreover, the single pass had 62.8% selectivity and 70.3 mol h^−1^ g_cat_^−1^ activity at 300 mA cm^−2^ industrial current, and the instantaneous concentration of salt-free H_2_O_2_ was 9.4 mm (Figure 5d). The stability of CoN@OCNT was examined under a constant current density of 60 mA cm^−2^ and exhibited < 5% change in H_2_O_2_ FE or yield over 80 h of operation; the cell voltage of reaction for 10 h and 80 h increased by only 0.08 v (Figure 5e and Figure S24). The excellent performance of CoN@OCNT was compared with the catalyst in a scalable electrolytic cell in a neutral medium at 60 mA cm^−2^ activity, which has high activity, high selectivity and high stability (Figure 5f and Table S5).

## 4. Conclusions

We developed a CoN@OCNT single-atom catalyst featuring spatially confined Co–NC coordination sites by pyrolyzing a well-defined CoL_6_ precursor anchored onto oxidized carbon nanotubes (OCNTs). This strategy enables precise regulation of the active-site configuration through controlled pyrolysis temperature, effectively overcoming the structural heterogeneity commonly encountered in conventional M–N–C catalysts. Benefiting from the optimized coordination environment, the CoN@OCNT catalyst exhibits a high onset potential of 0.66 V versus RHE and nearly 100% H_2_O_2_ selectivity over a broad potential range from −1.0 to 0.66 V versus RHE in neutral electrolyte. Moreover, the catalyst achieves an H_2_O_2_ production rate of 21.8 mol h^−1^ g_cat_^−1^ with excellent operational stability over 80 h in a scalable membrane electrode assembly (MEA) electrolyzer at 60 mA cm^−2^. Impressively, under an industrially relevant current density of 300 mA cm^−2^, the instantaneous H_2_O_2_ production rate reaches 70.3 mol h^−1^ g_cat_^−1^, accompanied by a salt-free H_2_O_2_ concentration of up to 9.4 mM and 62.8% FE for H_2_O_2_ production. These results demonstrate the strong potential of this catalyst system for practical, sustainable, and large-scale neutral H_2_O_2_ electrosynthesis.

## Data Availability

The data supporting the findings of this study are available within the article and in Supplementary Information Files. All data are available from the authors upon request.

## Supplementary Materials

The following supporting information can be downloaded at: https://www.mdpi.com/article/10.3390/nano16130813/s1. Figure S1: Morphology characterization of CNT and OCNT; Figure S2. Raman spectra of CNT and OCNT, greater D peak integration suggests more carbon defect; Figure S3. Morphology characterization of CoL6 and CoN@OCNT; Figure S4. Morphology characterization of CoN@OCNT; Figure S5. HAADF-STEM and EDS mapping (C, N, O, and Co) of CoN@OCNT; Figure S6. Morphology characterization of N@OCNT, Co@OCNT and CoN@CNT; Figure S7. (a) XRD patterns of CoL6, OCNT, CoN@OCNT and CoN@OCNT. (b) XPS O 1s spectra for CoN@OCNT; Figure S8. Structural characterization of CoN@OCNT at different pyrolysis temperatures; Figure S9. CV curves of CoL6, OCNT and CoN@OCNT in O_2_-saturated 0.1 M LiClO_4_ at 1600 rpm; Figure S10. Corresponding calculated electron transfer number over the applied potential range 0.0 V to 0.7 V vs. RHE; Figure S11. Comparison of mass activity measured at 0.5 V vs. RHE; Figure S12. Tafel plots of CoL6, OCNT, N@OCNT, Co@OCNT and CoN@OCNT; Figure S13. Effect of pyrolysis temperature on performance; Figure S14. The TOF values of the catalysts at different pyrolysis temperatures at 0.5 V vs. RHE were measured; Figure S15. The effect of synthesis method on performance; Figure S16. Co 2p high-resolution XPS spectra of CoN@OCNT, CoL6 and CoN@OCNT-GX; Figure S17. In-situ characterization device diagram; Figure S18. Net rates of H_2_O_2_ production on CoN@OCNT catalysts are expected to correlate to j_peroxide_ − j_PRR_; Figure S19. Electrochemical characterization of CoN@OCNT; Figure S20. The effect of different cathode flow rates on CoN@OCNT loading of 50 μg cm^−2^ was studied; Figure S21. The effect of different cathode flow rates on CoN@OCNT loading of 100 μg cm^−2^ was studied by MEA; Figure S22. The effect of load is tested by RRDE; Figure S23. The LSV curves of O_2_ and Ar in the membrane electrode with 50 μg cm^−2^ loading at 200 sccm, respectively; Figure S24. Long-term H_2_O_2_ electrosynthesis at 60 mA cm^−2^ in a MEA device; Figure S25. SEM image of CoN@OCNT. Table S1: XPS elemental quantification of OCNT, CoL_6_, N@OCNT, Co@OCNT, CoN@CNT, CoN@OCNT, CoN@OCNT-600, CoN@OCNT-800 and CoN@OCNT-GX; Table S2. The detailed XPS fitting parameters of Co 2p XPS spectra of samples; Table S3. The detailed XPS fitting parameters of N 1s XPS spectra of samples; Table S4. Summary of H_2_O_2_ electrocatalysts prepared by RRDE in neutral media; Table S5. Summary of preparation of H_2_O_2_ electrocatalyst in neutral medium by scalable electrolytic cell membrane-electrode-assembly cells (MEAs), gas diffusion electrode cells (GDEs), H type of electrolytic cell (H-cells), solid electrolyte cells (SEs).
