# Supplementary material for: Spatially Confined Co-N4 Sites on N-Doped Carbon Nanotube for Efficient Salt-Free Neutral H2O2 Electrosynthesis
Source: Nanomaterials (Basel). 2026 Jul 1;16(13):813. doi: 10.3390/nano16130813 (PMC13362725; doi:10.3390/nano16130813)
Supplement: Supplementary file 1 [file nanomaterials-16-00813-s001.zip › nanomaterials-4381066-supplementary.pdf]

Supporting Information

Spatially Confined Co-N_4 Sites on N-Doped Carbon Nanotube for Efficient Salt-Free Neutral H_2O_2 Electrosynthesis

Manman Zou †, Xiaoling Zhuang †, Qin Tian and Jili Yuan *

College of Materials & Metallurgy, Guizhou University, Huaxi District, Guiyang 550025, China; 13067288995@163.com (X.Z.)

* Correspondence: jlyuan@gzu.edu.cn

† These authors contributed equally to this work.

Supplementary Figure

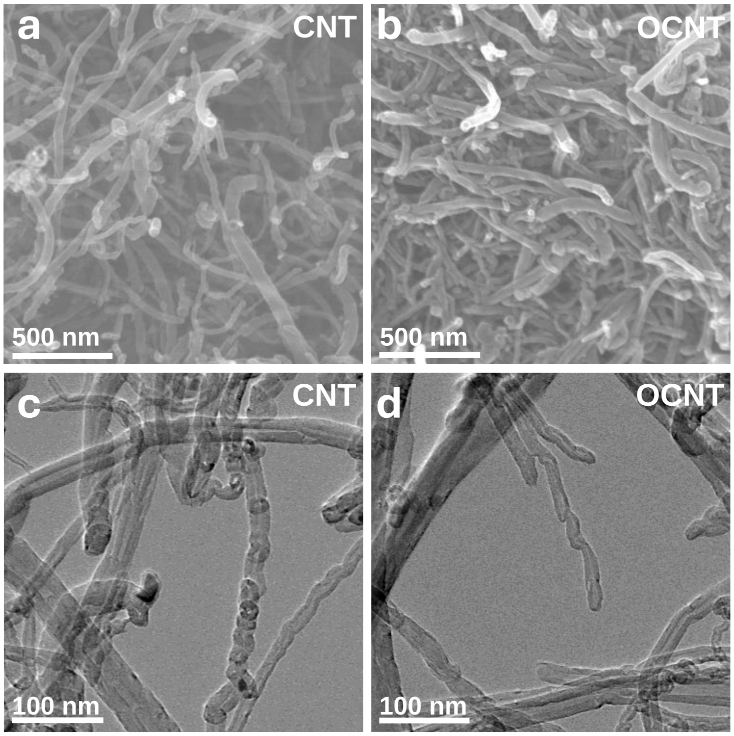

Figure S1. Morphology characterization of CNT and OCNT. a SEM image of CNT. **b** SEM image of OCNT. **c** TEM image of CNT. **d** TEM image of OCNT.

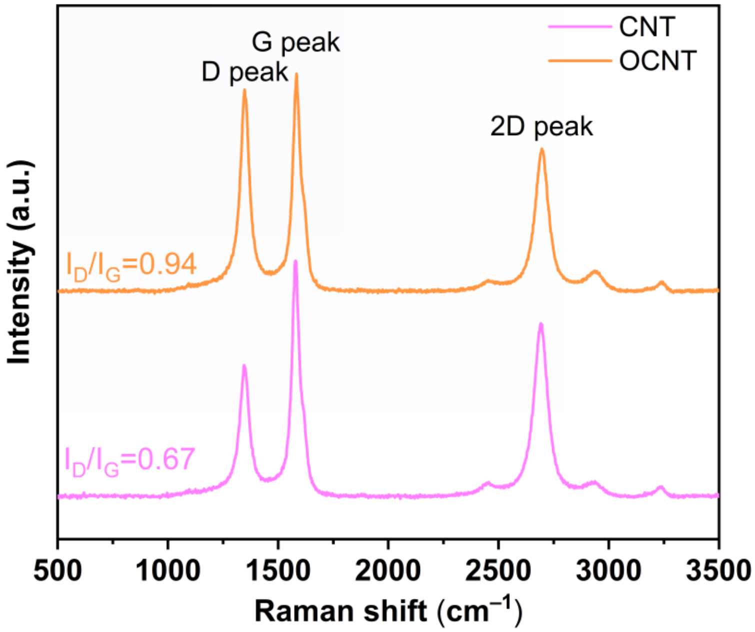

Figure S2. Raman spectra of CNT and OCNT, greater D peak integration suggests more carbon defect.

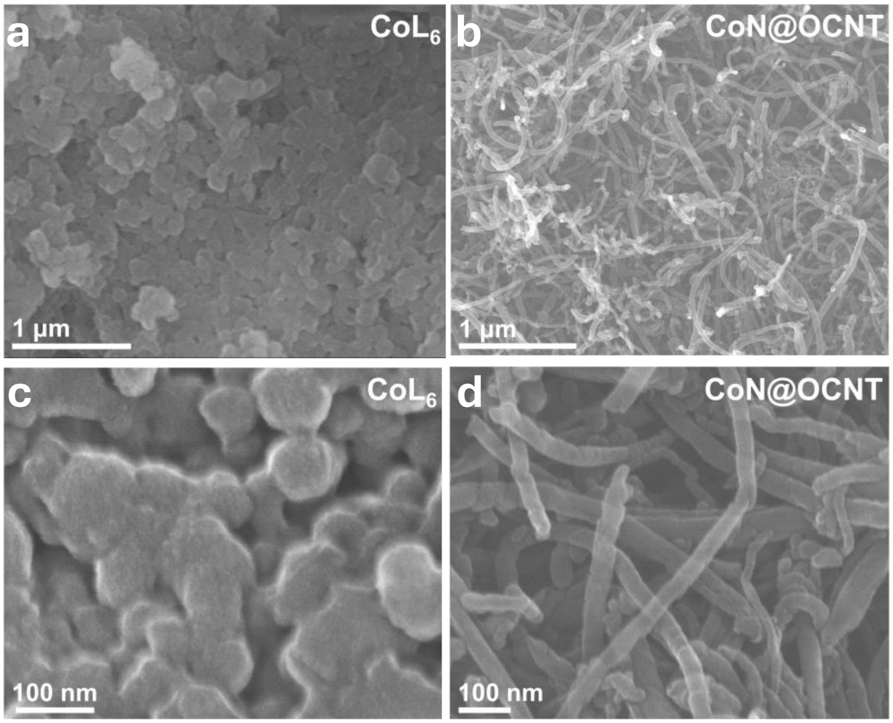

Figure S3. Morphology characterization of CoL_6 and CoN@OCNT. a, c SEM image of CoL_6. **b, d** SEM image of CoN@OCNT.

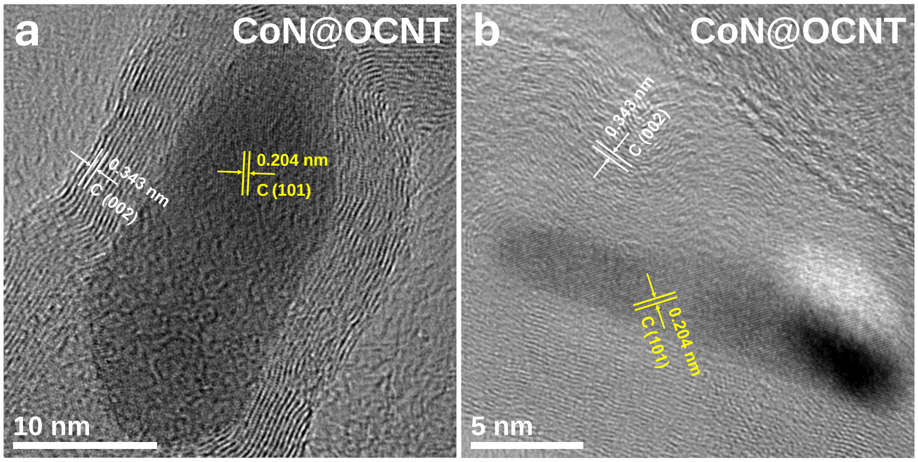

Figure S4. Morphology characterization of CoN@OCNT. a,b HR-TEM image of CoN@OCNT

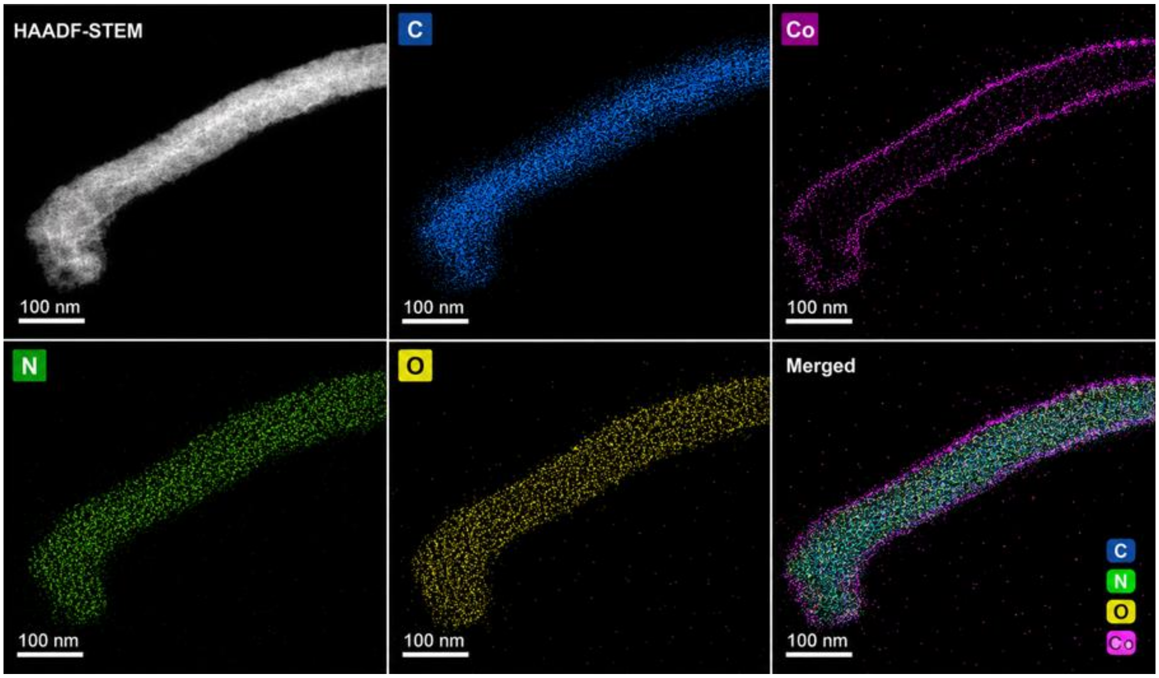

Figure S5. HAADF-STEM and EDS mapping (C, N, O, and Co) of CoN@OCNT.

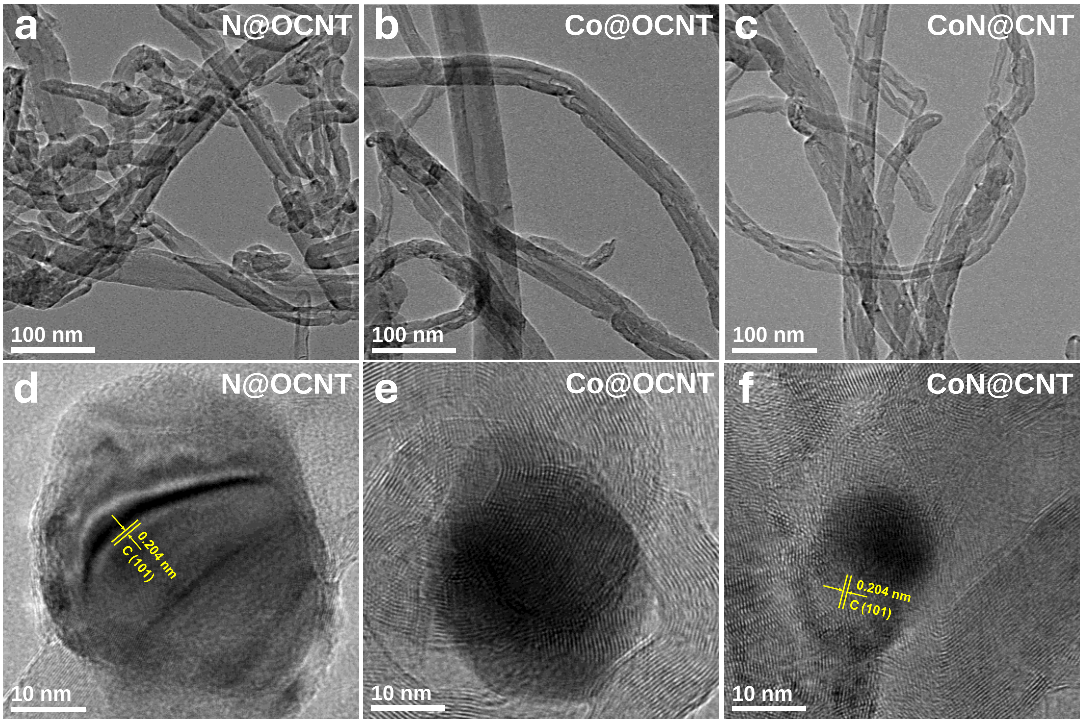

Figure S6. Morphology characterization of N@OCNT, Co@OCNT and CoN@CNT. a TEM image of N@OCNT. **b** TEM image of Co@OCNT. **c** TEM image of CoN@CNT. **d** HR-TEM image of N@OCNT. **e** HR-TEM image of Co@OCNT. **f** HR-TEM image of CoN@CNT.

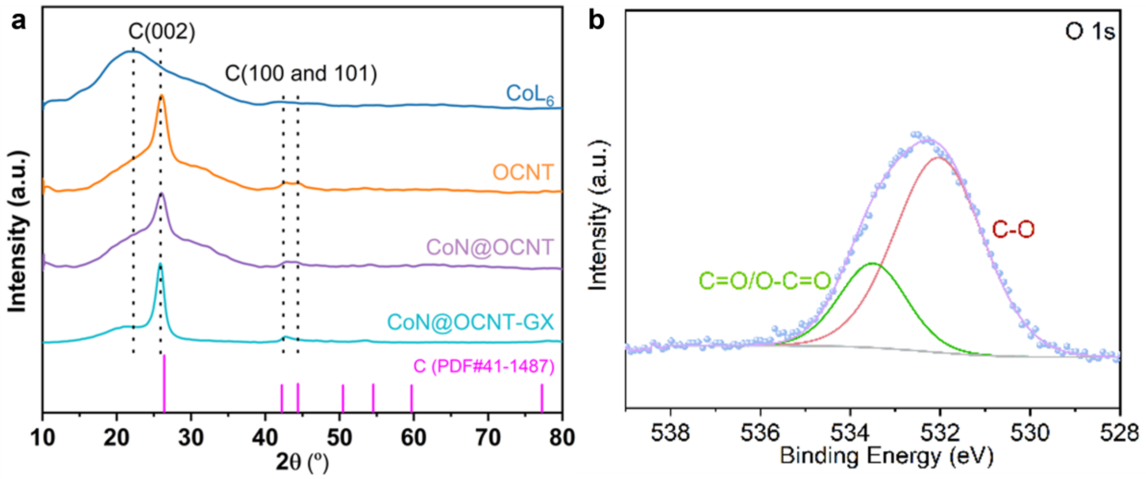

Figure S7. (a) XRD patterns of CoL_6, OCNT, CoN@OCNT and CoN@OCNT. (b) XPS O 1s spectra for CoN@OCNT.

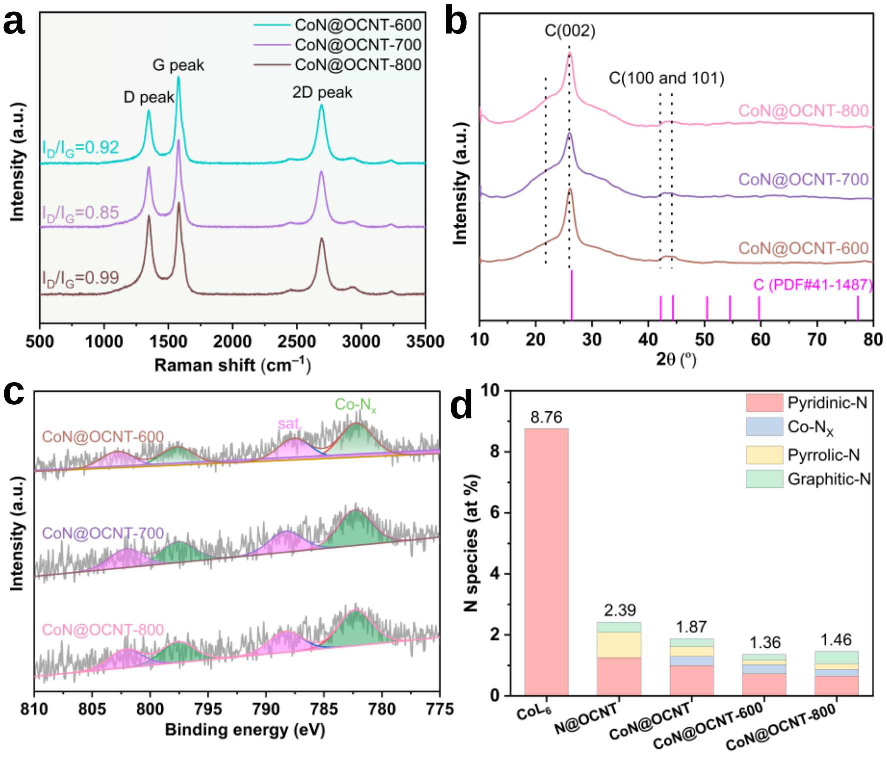

Figure S8. Structural characterization of CoN@OCNT at different pyrolysis temperatures. a Raman spectra of CoN@OCNT-600, CoN@OCNT-700 and CoN@OCNT-800. **b** XRD patterns of CoN@OCNT-600, CoN@OCNT-700 and CoN@OCNT-800. **c** Co 2p high-resolution XPS spectra of CoN@OCNT-600, CoN@OCNT-700 and CoN@OCNT-800. **d** N species of CoL_6, N@OCNT,

CoN@OCNT-600, CoN@OCNT-700 and CoN@OCNT-800.

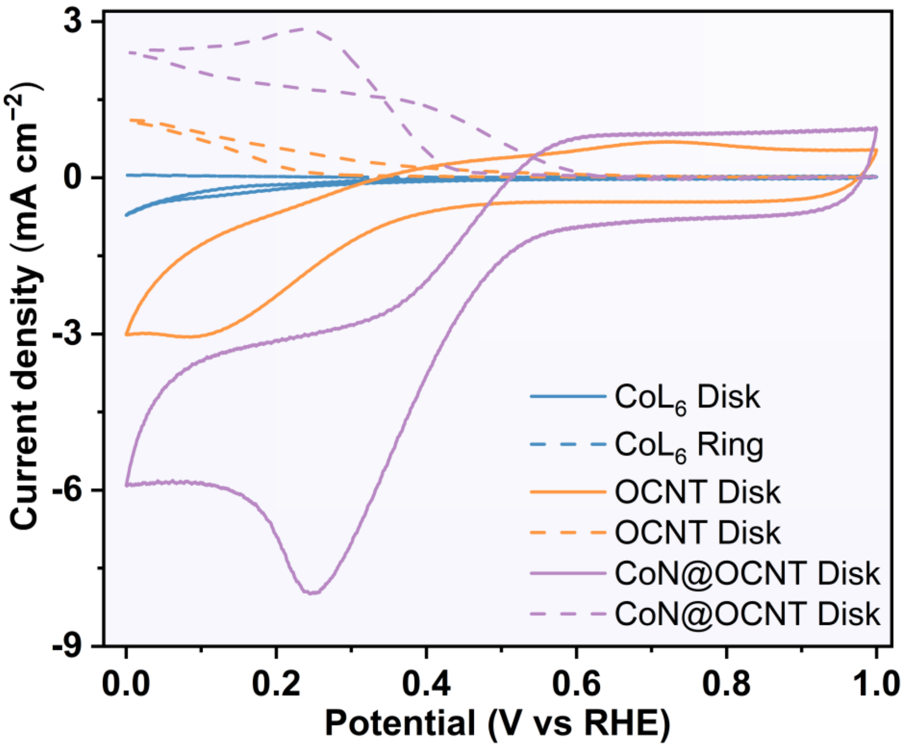

Figure S9. CV curves of CoL_6, OCNT and CoN@OCNT in O_2-saturated 0.1M $LiClO_4$ at 1600 rpm. (Upper: ring current densities; Bottom: Disk current densities).

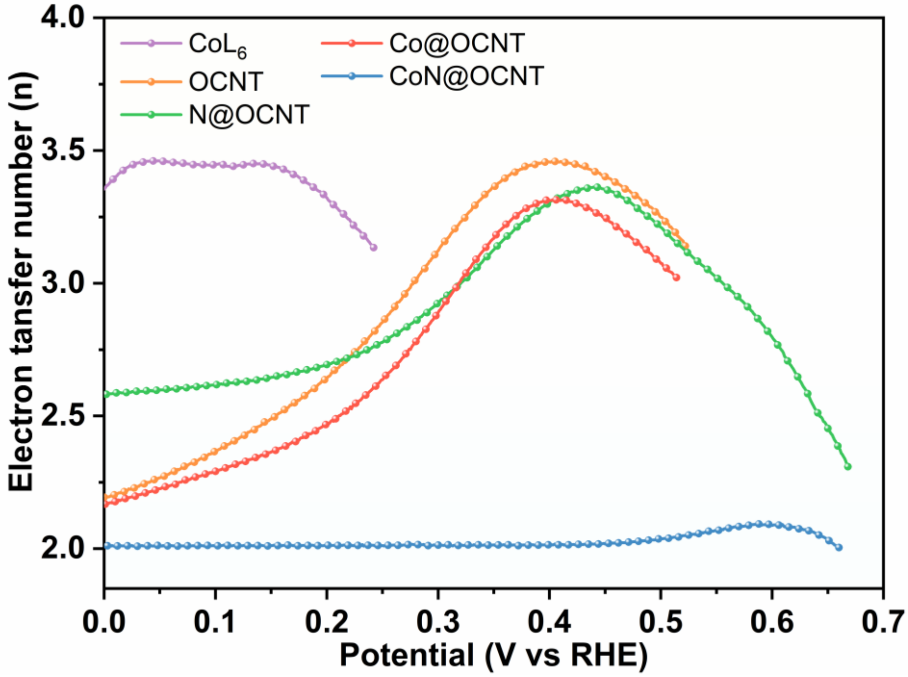

Figure S10. Corresponding calculated electron transfer number over the applied potential range 0.0 V to 0.7 V vs RHE.

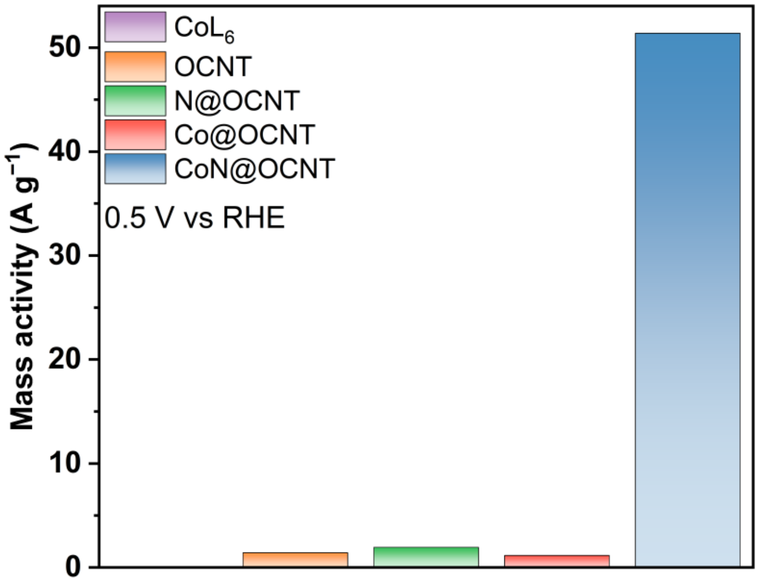

Figure S11. Comparison of mass activity measured at 0.5 V vs RHE.

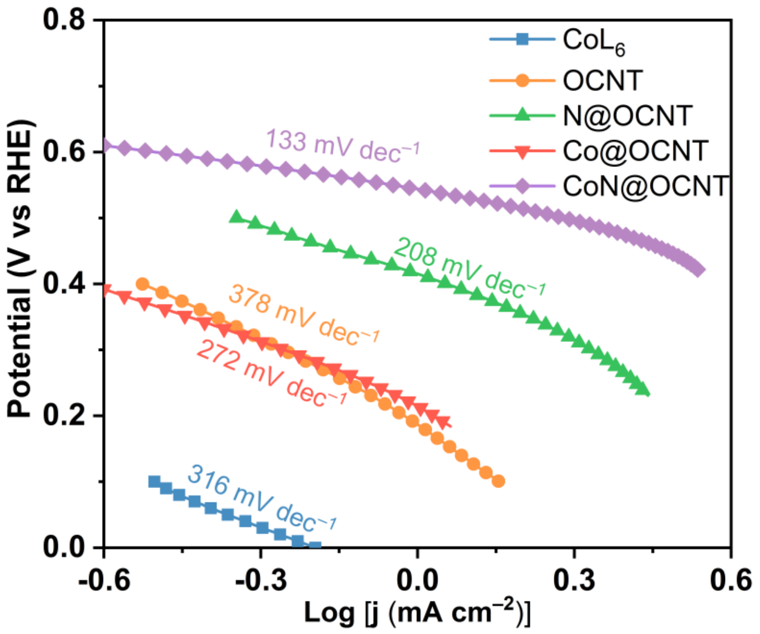

Figure S12. Tafel plots of CoL_6, OCNT, N@OCNT, Co@OCNT and CoN@OCNT.

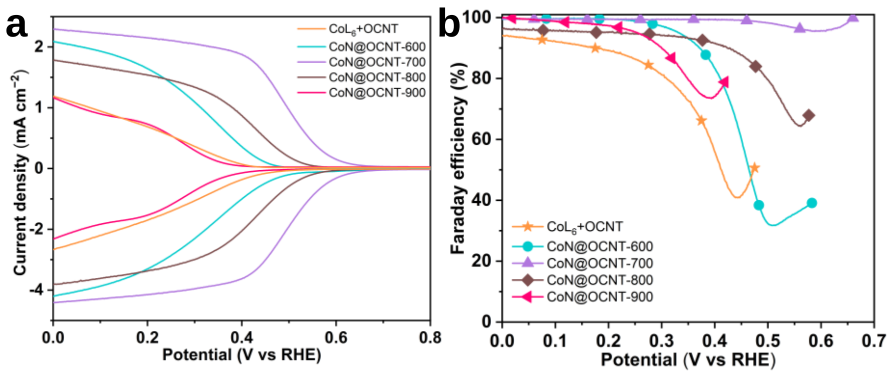

Figure S13. Effect of pyrolysis temperature on performance. a Comparison of LSV curves of different catalysts in O_2-saturated 0.1M $LiClO_4$ at 1600 rpm. **b** H_2O_2 FE over the applied potential range 0.0 V to 0.7 V vs RHE.

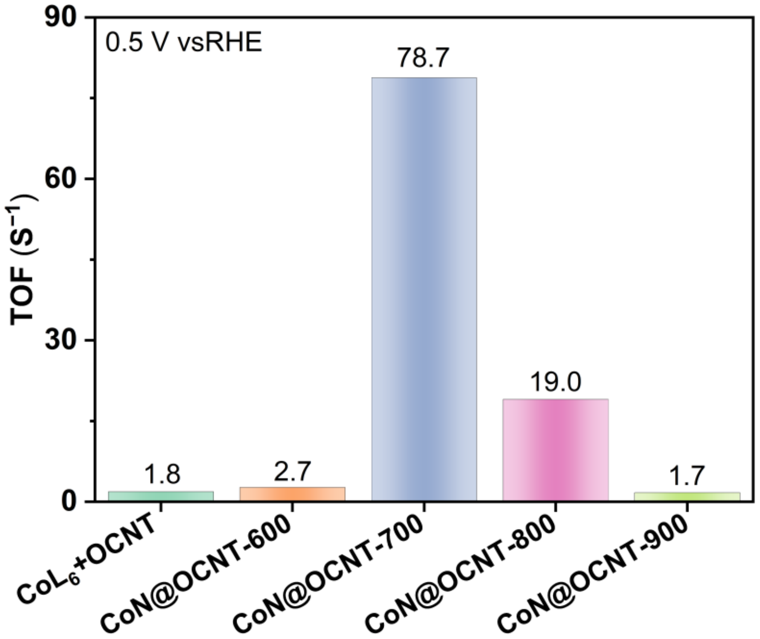

Figure S14. The TOF values of the catalysts at different pyrolysis temperatures at 0.5 V vs RHE were measured.

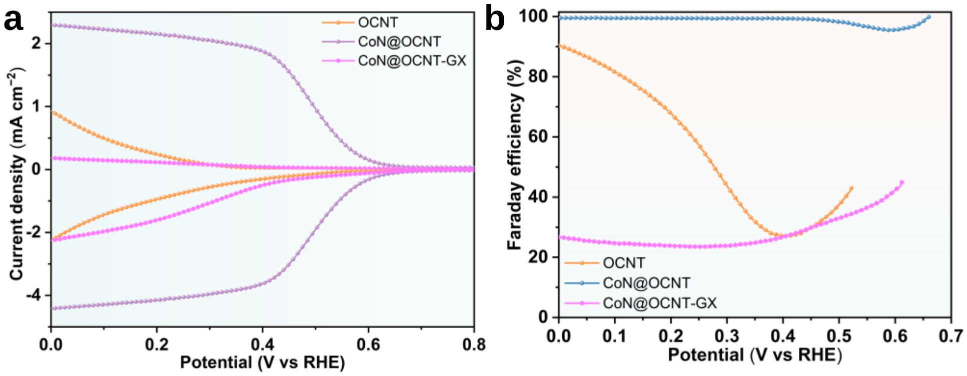

Figure S15. The effect of synthesis method on performance. a Comparison of LSV curves of different catalysts in O_2-saturated 0.1M $LiClO_4$ at 1600 rpm. **b** H_2O_2 FE over the applied potential range 0.0 V to 0.7 V vs RHE.

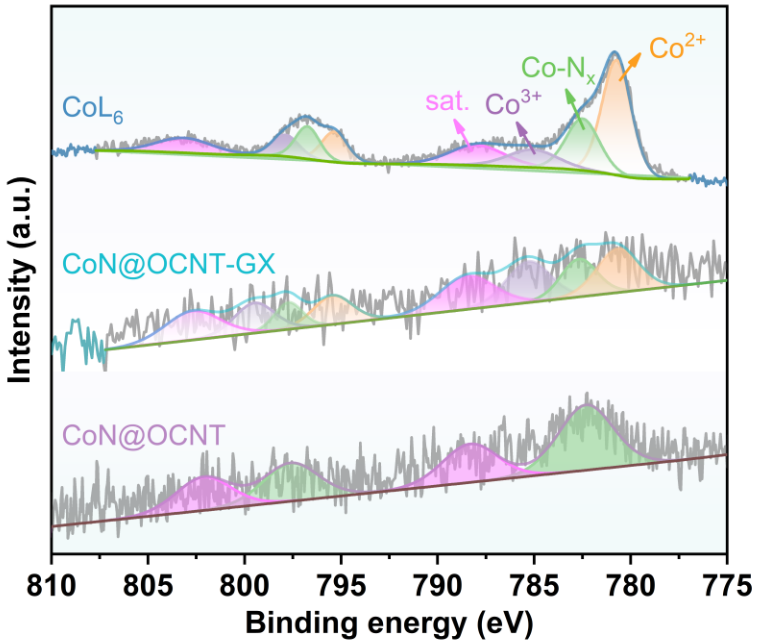

Figure S16. Co 2p high-resolution XPS spectra of CoN@OCNT, CoL_6 and CoN@OCNT-GX.

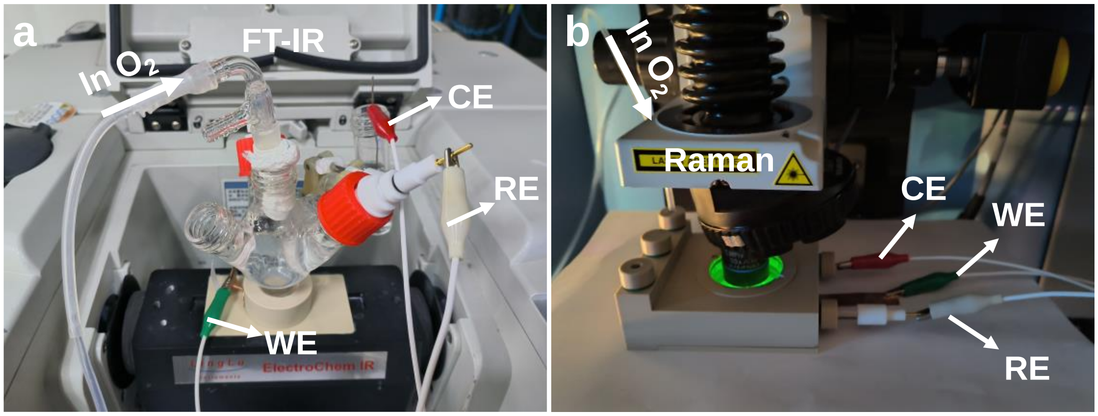

Figure S17. In-situ characterization device diagram. a In-situ FT-IR device diagram. **b** In-situ Raman device diagram.

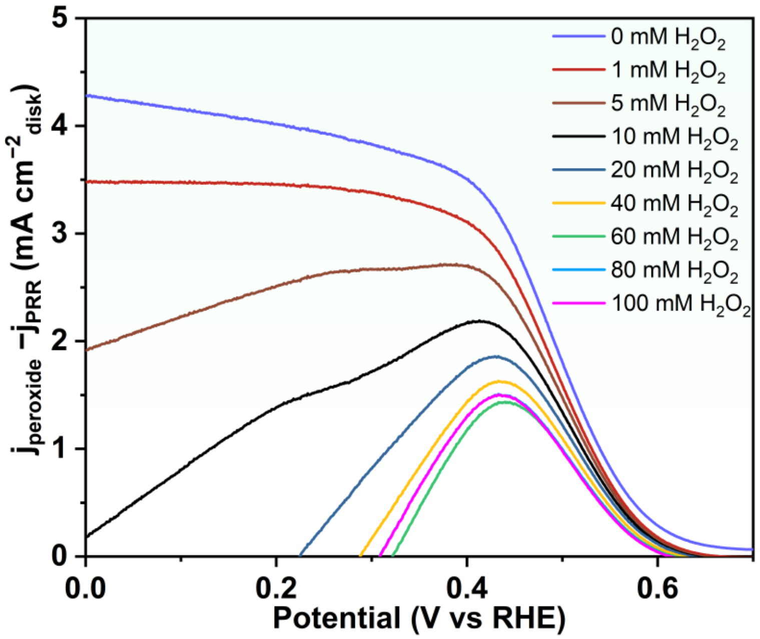

Figure S18. Net rates of H_2O_2 production on CoN@OCNT catalysts are expected to correlate to $j_{peroxide} - j_{PRR}$.

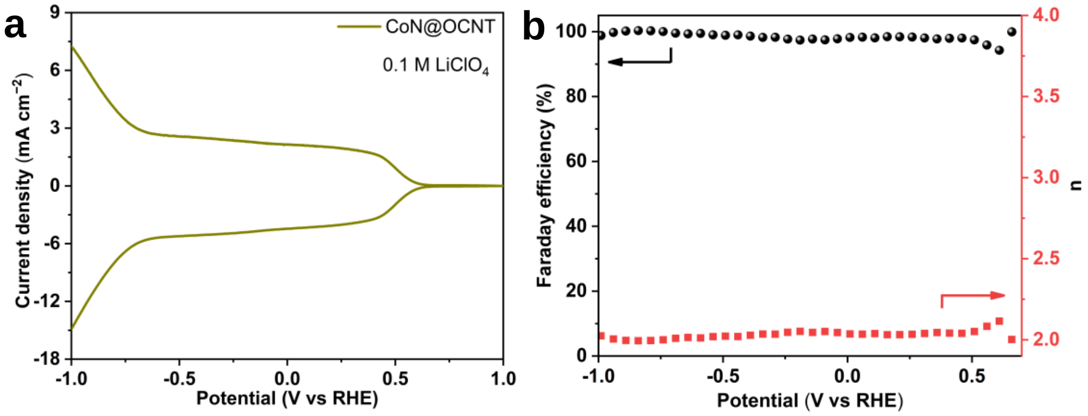

Figure S19. Electrochemical characterization of CoN@OCNT. a The LSV curve of CoN@OCNT at wide potential. **b** The calculated FE and n are in the range of-1.0 V to 0.7 V.

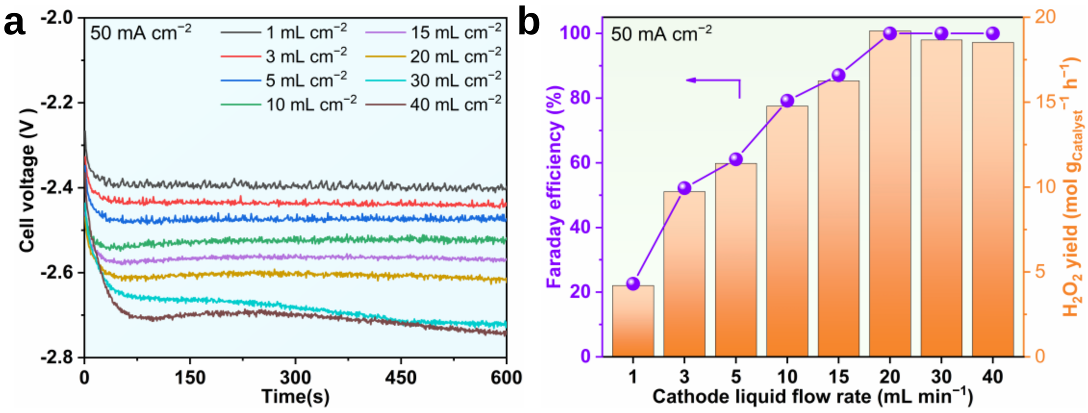

Figure S20. The effect of different cathode flow rates on CoN@OCNT loading of 50 μg cm^{-2} was studied. a The effect of different DI water flow rates on the cell voltage at the cathode 200 sccm O_2. **b** Effect of DI water flow rate on H_2O_2 selectivity and yield.

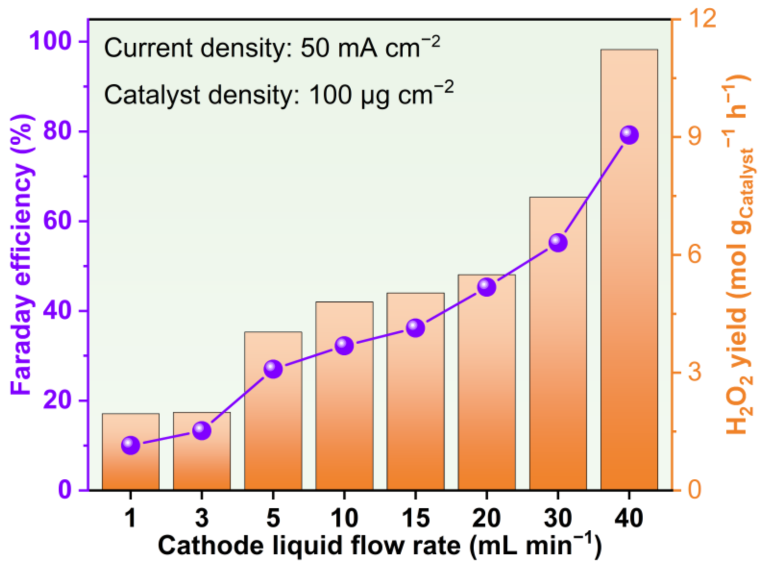

Figure S21. The effect of different cathode flow rates on CoN@OCNT loading of 100 μg cm^{-2} was studied by MEA.

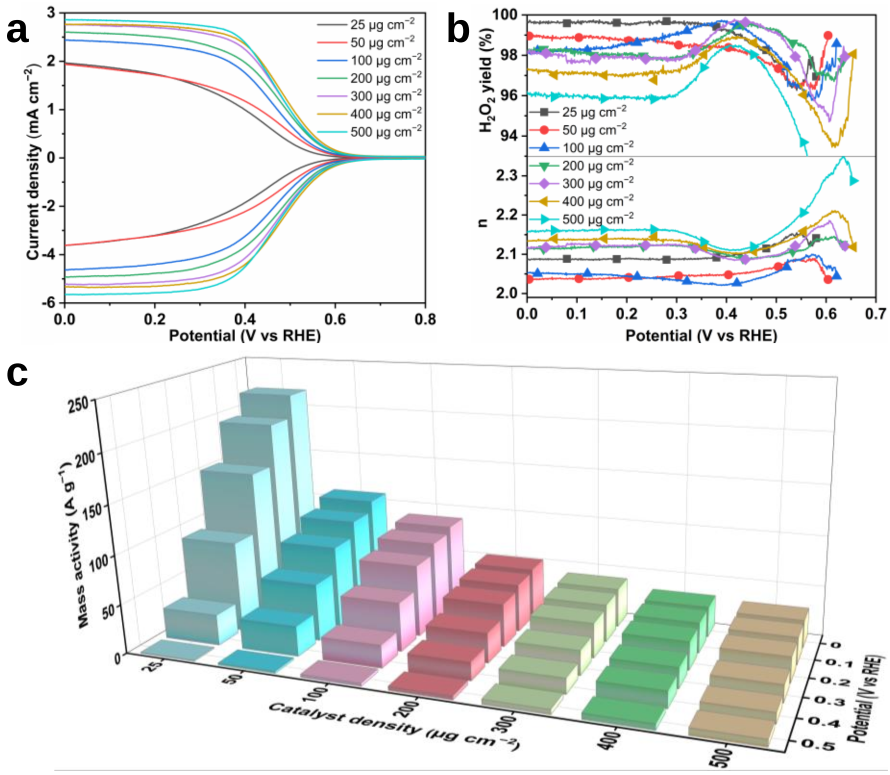

Figure S22. The effect of load is tested by RRDE. a LSV curves of CoN@OCNT at various catalyst loading in 0.1 M $LiClO_4$. **b** Calculated H_2O_2 selectivity and n. c The calculated mass activity at different potentials.

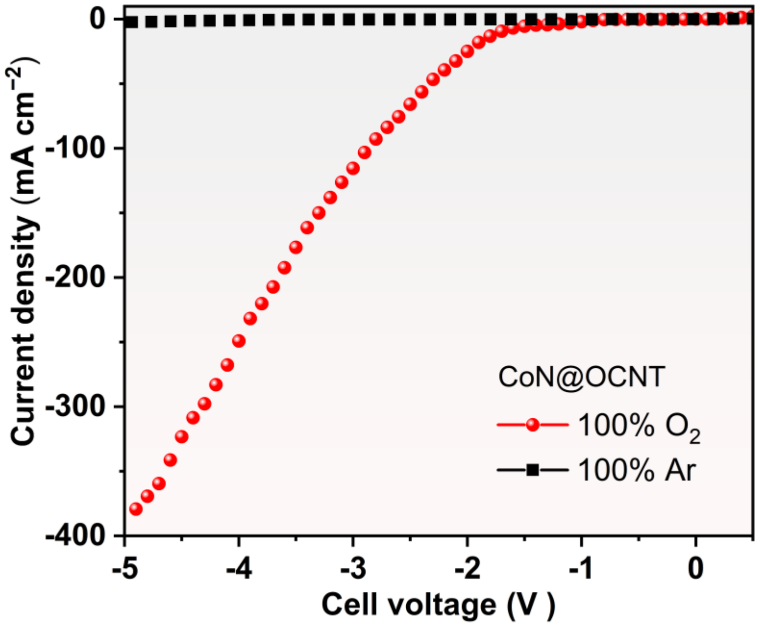

Figure S23. The LSV curves of O_2 and Ar in the membrane electrode with 50 μg cm^{-2} loading at 200 sccm, respectively.

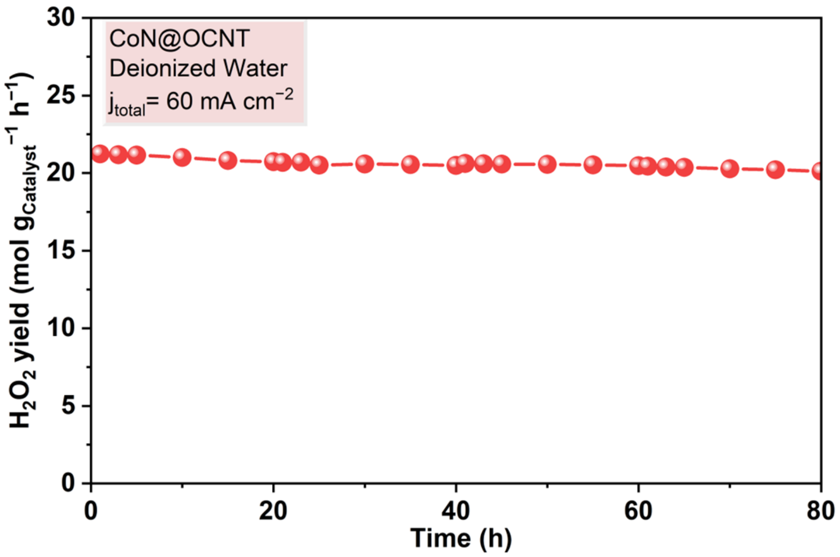

Figure S24. Long-term H_2O_2 electrosynthesis at 60 mA cm^{-2} in a MEA device.

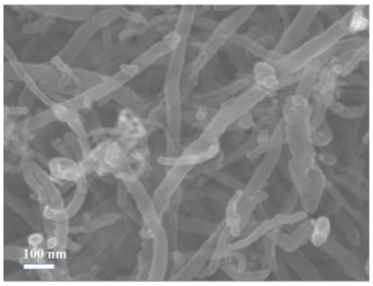

Figure S25. SEM image of CoN@OCNT

Supplementary Tables

Table S1. XPS elemental quantification of OCNT, CoL_6, N@OCNT, Co@OCNT, CoN@CNT, CoN@OCNT, CoN@OCNT-600, CoN@OCNT-800 and CoN@OCNT-GX.

Sample	C (at%)	N (at%)	O (at%)	Co (at%)	F (at%)
OCNT	98.53	----	1.47	----	----
CoL_6	74.13	7.40	----	1.20	17.27
N@OCNT	94.53	2.39	3.08	----	----
Co@OCNT	97.63	----	1.97	0.40	----
CoN@CNT	96.05	1.45	2.33	0.16	0.20
CoN@OCNT	93.49	3.23	2.73	0.23	0.32
CoN@OCNT-600	95.85	1.41	2.41	0.11	0.21
CoL_6@OCNT-800	95.40	1.82	2.41	0.14	0.24
CoN@OCNT-GX	96.34	1.52	1.81	0.11	0.22

Table S2. The detailed XPS fitting parameters of Co 2p XPS spectra of samples.

Species	CoL_6	Co@OCNT	CoN@OCNT	CoN@OCNT-600	CoN@OCNT-800	CoN@OCNT-GM
Co^{2+} $2P_{3/2}$	780.7	781.2	----	----	----	780.7
$Co\text{-}N_X$ $2P_{3/2}$	782.4	----	782.3	782.2	781.6	782.7
Co^{3+} $2P_{3/2}$	785.1	784.9	----	----	----	785.3
Co^{2+} $2P_{1/2}$	795.4	796.9	----	----	----	795.4
$Co\text{-}N_X$ $2P_{1/2}$	796.7	----	797.6	797.7	797.0	797.7
Co^{3+} $2P_{1/2}$	797.7	800.6	----	----	----	799.5

Table S3. The detailed XPS fitting parameters of N 1s XPS spectra of samples.

Species	CoL_6	N@OCNT	CoN@OCNT	CoN@CNT	CoN@OCNT-600	CoN@OCNT-800	CoN@OCNT-GX
Pyridinic-N	399.1	398.8	398.9	398.9	399.0	398.8	398.8
	100.0 %	52.4 %	52.9 %	44.4 %	53.2 %	44.4 %	48.3 %
$Co\text{-}N_X$	----	----	400.0	400.0	400.0	400.0	400.0

	----	----	16.4 %	18.2 %	22.3 %	15.1 %	10.6 %
Pyrrolic-N	----	400.7	400.8	400.7	400.7	400.8	400.8
	----	34.7 %	16.9 %	19.8 %	10.6 %	12.9 %	15.5 %
Graphitic-N	----	401.8	401.8	401.7	401.8	401.8	401.8
	----	13.9 %	13.8 %	19.6 %	13.9 %	27.6 %	25.6 %

Table S4. Summary of H_2O_2 electrocatalysts prepared by RRDE in neutral media

**Catalyst**	**Electrolyte**	**Onset potentials**	**$J_{k, H2O2}$ = 1 mA/cm$^2_{disk}$**	**Stability test**	**Ref.**
CoN@OCNT	0.1 M $LiClO_4$	0.65 V	97.0%@0.55	18 h	This work
CoPc-6wt%/o-SWCNT-2	0.1 M K_2SO_4	0.45 V	93.1%@0.40	--	[1]
$ZnO@ZnO_2$	0.1 M K_2SO_4	0.45 V	91.5%@0.38	40 h	[2]
h-SnO_2	0.1 M Na_2SO_4	0.50 V	97.9%@0.37	--	[3]
ZnCo-ZIF	0.1 M PBS	0.59 V	90.4%@0.40	2 h	[4]
N-C_{800}	0.5 M NaCl	0.61 V	90.0%@0.45	10 h	[5]
NiPyCN/CN	0.1 M K_2SO_4	0.50 V	90.8%@0.45	16 h	[6]
CoPC-CNT(O)	0.1 M K_2SO_4	0.58 V	94.6%@0.46	10 h	[7]
Co-N-C	0.1 M PBS	0.80 V	55.1%@0.72	10 h	[8]
ZnO-v	0.6 M K_2SO_4	0.43 V	92.8%@0.35	--	[9]
L-ZnO	0.6 M K_2SO_4	0.35 V	97.4%@0.05	--	[10]
CoPC-OCNT	0.1 M K_2SO_4	0.60 V	93@0.50	30 h	[11]
Bi/NPC	0.05M Na_2SO4	---	---	2 h	[12]
C-$CoSe_2$	0.05 M NiPi	0.75 V	80%@0.6	Cycle 250	[13]
Ni-HAB	0.05 M NaPi	0.62 V	60%@0.50	1.5 h	[14]
MBC-2	0.05 M Na_2SO_4	0.46 V	84.5%@0.31	6 h	[15]
Pd_4Se NPs	0.1 M KCl	0.71 V	80%@0.60	2 h	[16]
Co–SAs/NC	0.1 M Na_2SO_4	0.48 V	30%@0.15	NA	[17]
B-C	0.1 M Na_2SO_4	0.45 V	79%@0.30	NA	[18]
PCMNS	0.1 M K_2SO_4	0.49 V	87.9%@0.41	10 h	[19]

MCHS	0.1 M PBS	0.61 V	90%@0.56	Cycle 2500	[20]
CB600	0.1 M Na_2SO_4	0.41 V	90%@0.38	0.5 h	[21]

Table S5. Summary of preparation of H_2O_2 electrocatalyst in neutral medium by scalable electrolytic cell membrane-electrode-assembly cells (MEAs), gas diffusion electrode cells (GDEs), H type of electrolytic cell (H-cells), solid electrolyte cells (SEs)

**Catalyst**	**Electrolyte**	**Electrolytic cell**	**Catalyst loading (μg/cm^2)**	**H_2O_2 production rate mol $g_{catalyst}^{-1}$ h^{-1}**	**FE or H_2O_2 selectivity**	**Stability test**	**Ref.**
CoN@OCNT	DI water	MEAs	50	21.60	91.0%	80 h	This work
CoPc-6wt%/o-SWCNT-2	0.1 M K_2SO_4	H-cell	100	3.30	90.0%	75 h	[1]
ZnO@ZnO_2	0.1 M K_2SO_4	GDEs	400	5.47	95.5%	50 h	[2]
h-SnO_2	1.0 M Na_2SO_4	GDEs	530	3.89	90.0%	40 h	[3]
w/TS-1	DI water	SEs	369.8	--	96.0%	5 h	[22]
ZnCo-ZIF	0.1 M PBS	GDEs	610	4.30	99.0%	100 h	[4]
N-C_{800}	0.5 M NaCl	H-cell	--	0.63	79.8%	5.5 h	[5]
NiPyCN/CN	0.1 M K_2SO_4	H-cell	500	3.89	80.5%	80 h	[6]
CoPC-CNT(O)	1 M Na_2SO_4	GDEs	200	26.1	38.0%	100 h	[7]
Co-N-C	0.5 M PBS	GDEs	400	---	----	3 h	[8]
ZnO-v	0.6 M K_2SO_4	GDEs	5000	3.66	98.1%	20 h	[9]
L-ZnO	0.6 M K_2SO_4	GDEs	5000	3.47	93.2%	20 h	[10]
CoPC-OCNT	0.1 M K_2SO_4	GDEs	500	0.47	92.0%	2.5 h	[11]

Bi/NPC	0.5M Na_2SO4	GDEs	100	5.29	71.2%	2 h	[12]
C-$CoSe_2$	0.05 M NaPi	H-cell	200	---	---	5 h	[13]
Ni-HAB	0.05 M NaPi	GDEs	100	12.9	39.5%	1.5 h	[14]
MBC-2	0.05 M Na_2SO4	--	220	6.4	80.0%	2 h	[15]
Pd_4Se NPs	0.1 M KCl	MEAs	500	6.0	79.2%	2 h	[16]
Co–SAs/NC	0.1 M Na_2SO_4	SEs	1000	0.06	90.0%	10 h	[17]
B-C	1 M Na_2SO_4	--	500	4.6	84.5%	30 h	[18]
PCMNS	0.1 M K_2SO_4	H-cell	500	1.1	80.5%	10 h	[19]
MCHS	0.1 M PBS	--	100	0.03	93.1%	1 h	[20]
CB600	0.1 M Na_2SO_4	GDEs	500	0.3	76.3%	0.5 h	[21]

Supplementary References

1. Li, Y.; Cheng, H.; Wang, M.; Xu, J.; Guan, L., Highly Coordinative Molecular Cobalt-Phthalocyanine Electrocatalyst on Oxidized Single-Walled Carbon Nanotube for Efficient Hydrogen Peroxide Production. *Mater. Horiz.* **2024,** 11, (10), 2517-2527.
2. Zhou, Y.; Xu, L.; Wu, J.; Zhu, W.; He, T.; Yang, H.; Huang, H.; Cheng, T.; Liu, Y.; Kang, Z., The operation active site of O_2 reduction to H_2O_2 over ZnO. *Energy & Environmental Science* **2023,** 16, (8), 3526-3533.
3. Zhang, Y.; Wang, M.; Zhu, W.; Fang, M.; Ma, M.; Liao, F.; Yang, H.; Cheng, T.; Pao, C.-W.; Chang, Y.-C.; Hu, Z.; Shao, Q.; Shao, M.; Kang, Z., Metastable Hexagonal Phase SnO_2 Nanoribbons with Active Edge Sites for Efficient Hydrogen Peroxide Electrosynthesis in Neutral Media. *Angew. Chem. Int. Ed.* **2023,** 62, (20), e202218924.
4. Zhang, C.; Yuan, L.; Liu, C.; Li, Z.; Zou, Y.; Zhang, X.; Zhang, Y.; Zhang, Z.; Wei, G.; Yu, C., Crystal Engineering Enables Cobalt-Based Metal-Organic Frameworks as High-Performance Electrocatalysts for H_2O_2 Production. *J. Am. Chem. Soc.* **2023,** 145, (14).
5. Wang, N.; Ma, S.; Zhang, R.; Wang, L.; Wang, Y.; Yang, L.; Li, J.; Guan, F.; Duan, J.; Hou, B., Regulating N Species in N-Doped Carbon Electro-Catalysts for High-Efficiency Synthesis of Hydrogen Peroxide in Simulated Seawater. *Adv. Sci.* **2023,** 10, (31), 2302446.
6. Sun, L.; Jin, X.; Su, T.; Fisher, A. C.; Wang, X., Conjugated Nickel Phthalocyanine Derivatives for Heterogeneous Electrocatalytic H_2O_2 Synthesis. *Adv. Mater.* **2023,** 36, (17), 2306336.
7. Lee, B.-H.; Shin, H.; Rasouli, A. S.; Choubisa, H.; Ou, P.; Dorakhan, R.; Grigioni, I.; Lee, G.; Shirzadi, E.; Miao, R. K.; Wicks, J.; Park, S.; Lee, H. S.; Zhang, J.; Chen, Y.; Chen, Z.; Sinton, D.; Hyeon, T.; Sung, Y.-E.; Sargent, E. H., Supramolecular tuning of supported metal phthalocyanine catalysts for hydrogen peroxide electrosynthesis. *Nat. Catal.* **2023,** 6, 234-243.
8. Du, Y. X.; Yang, Q.; Lu, W. T.; Guan, Q. Y.; Cao, F. F.; Zhang, G., Carbon Black-Supported Single-Atom Co-N-C as an Efficient Oxygen Reduction Electrocatalyst for H_2O_2 Production in Acidic Media and Microbial Fuel Cell in Neutral Media. *Adv. Funct. Mater.* **2023,** 33, (27), 2300895.
9. Ding, S.; Zhang, Y.; Lou, F.; Li, M.; Huang, Q.; Yang, K.; Xia, B.; Tang, C.; Duan, J.; Antonietti, M.; Chen, S., Oxygen-vacancy-type Mars–van Krevelen mechanism drives ultrafast dioxygen electroreduction to hydrogen peroxide. *Mater. Today Energy* **2023,** 38, 101430.
10. Ding, S.; Xia, B.; Li, M.; Lou, F.; Cheng, C.; Gao, T.; Zhang, Y.; Yang, K.; Jiang, L.; Nie, Z.; Guan, H.; Duan, J.; Chen, S., An abnormal size effect enables ampere-level O_2 electroreduction to hydrogen peroxide in neutral electrolytes. *Energy Environ. Sci.* **2023,** 16, (8), 3363-3372.
11. Cao, P.; Quan, X.; Nie, X.; Zhao, K.; Liu, Y.; Chen, S.; Yu, H.; Chen, J. G., Metal single-site catalyst design for electrocatalytic production of hydrogen

peroxide at industrial-relevant currents. *Nat. Commun.* **2023,** 14, (1), 1-12.
12. Bao, Z.; Zhao, J.; Zhang, S.; Peng, X.; Shao, Y.; Jiang, C.; Xu, Z.; Zhong, X.; Yao, Z.; Wang, J., Tuning the ratio of Bi/Bi2O3 in Bi/PNC nanosheet for high-efficiency electrosynthesis hydrogen peroxide. *Nano Res.* **2023,** 16, (7), 9050-9058.
13. Sheng, H.; Ross, R. D.; Schmidt, J. R.; Jin, S., Metal-Compound-Based Electrocatalysts for Hydrogen Peroxide Electrosynthesis and the Electro-Fenton Process. *ACS Energy Lett.* **2022,** 8, 196-212.
14. Ross, R. D.; Sheng, H.; Ding, Y.; Janes, A. N.; Feng, D.; Schmidt, J. R.; Segre, C. U.; Jin, S., Operando Elucidation of Electrocatalytic and Redox Mechanisms on a 2D Metal Organic Framework Catalyst for Efficient Electrosynthesis of Hydrogen Peroxide in Neutral Media. *J. Am. Chem. Soc.* **2022,** 144, (34), 15845-15854.
15. Gao, M.; Wang, Z.-Y.; Yuan, Y.-R.; Li, W.-W.; Liu, H.-Q.; Huang, T.-Y., Ball-milled biochar for efficient neutral electrosynthesis of hydrogen peroxide. *Chem. Eng. J.* **2022,** 434, 134788.
16. Yang, C.; Bai, S.; Yu, Z.; Feng, Y.; Huang, B.; Lu, Q.; Wu, T.; Sun, M.; Zhu, T.; Cheng, C.; Zhang, L.; Shao, Q.; Huang, X., A newly-explored Pd-based nanocrystal for the pH-universal electrosynthesis of H_2O_2. *Nano Energy* **2021,** 89, 106480.
17. Xu, H.; Zhang, S.; Geng, J.; Wang, G.; Zhang, H., Cobalt single atom catalysts for the efficient electrosynthesis of hydrogen peroxide. *Inorg. Chem. Front.* **2021,** 8, (11), 2829-2834.
18. Xia, Y.; Zhao, X.; Xia, C.; Wu, Z. Y.; Zhu, P.; Kim, J. Y. T.; Bai, X.; Gao, G.; Hu, Y.; Zhong, J.; Liu, Y.; Wang, H., Highly active and selective oxygen reduction to H_2O_2 on boron-doped carbon for high production rates. *Nat. Commun.* **2021,** 12, (1), 4225.
19. Hu, R.; Cui, Y.; Huang, B.; Guan, L., Transforming C(60) Molecules into Polyhedral Carbon Micro-Nano Shells for Electrochemically Producing H_2O_2 in Neutral Electrolytes. *ACS Appl. Mater. Interfaces* **2021,** 13, (30), 35856-35864.
20. Pang, Y.; Wang, K.; Xie, H.; Sun, Y.; Titirici, M.-M.; Chai, G.-L., Mesoporous Carbon Hollow Spheres as Efficient Electrocatalysts for Oxygen Reduction to Hydrogen Peroxide in Neutral Electrolytes. *ACS Catal.* **2020,** 10, (14), 7434-7442.
21. Zhang, H.; Li, Y.; Zhao, Y.; Li, G.; Zhang, F., Carbon Black Oxidized by Air Calcination for Enhanced H_2O_2 Generation and Effective Organics Degradation. *ACS Appl. Mater. Interfaces* **2019,** 11, (31), 27846-27853.
22. Zhang, S.-K.; Feng, Y.; Elgazzar, A.; Xia, Y.; Qiu, C.; Adler, Z.; Sellers, C.; Wang, H., Interfacial electrochemical-chemical reaction coupling for efficient olefin oxidation to glycols. *Joule* **2023,** 7, (8), 1887-1901.
